# Macroalgal virosphere assists with host–microbiome equilibrium regulation and affects prokaryotes in surrounding marine environments

**DOI:** 10.1093/ismejo/wrae083

**Published:** 2024-05-06

**Authors:** Jiulong Zhao, Shailesh Nair, Zenghu Zhang, Zengmeng Wang, Nianzhi Jiao, Yongyu Zhang

**Affiliations:** Qingdao Institute of Bioenergy and Bioprocess Technology, Chinese Academy of Sciences, Qingdao, 266101, China; Shandong Energy Institute, Qingdao, Shandong, 266101, China; Qingdao New Energy Shandong Laboratory, Qingdao, 266101, China; Qingdao Institute of Bioenergy and Bioprocess Technology, Chinese Academy of Sciences, Qingdao, 266101, China; Shandong Energy Institute, Qingdao, Shandong, 266101, China; Qingdao New Energy Shandong Laboratory, Qingdao, 266101, China; Qingdao Institute of Bioenergy and Bioprocess Technology, Chinese Academy of Sciences, Qingdao, 266101, China; Shandong Energy Institute, Qingdao, Shandong, 266101, China; Qingdao New Energy Shandong Laboratory, Qingdao, 266101, China; University of Chinese Academy of Sciences, Beijing, 100049, China; Qingdao Institute of Bioenergy and Bioprocess Technology, Chinese Academy of Sciences, Qingdao, 266101, China; Shandong Energy Institute, Qingdao, Shandong, 266101, China; Qingdao New Energy Shandong Laboratory, Qingdao, 266101, China; Institute of Marine Microbes and Ecospheres, State Key Laboratory of Marine Environmental Science, Xiamen University, Xiamen, 361005, China; Qingdao Institute of Bioenergy and Bioprocess Technology, Chinese Academy of Sciences, Qingdao, 266101, China; Shandong Energy Institute, Qingdao, Shandong, 266101, China; Qingdao New Energy Shandong Laboratory, Qingdao, 266101, China; University of Chinese Academy of Sciences, Beijing, 100049, China

**Keywords:** kelp virosphere, endophytic/epiphytic virus, virus–prokaryote pairings, sediment virus, auxiliary metabolic gene

## Abstract

The microbiomes in macroalgal holobionts play vital roles in regulating macroalgal growth and ocean carbon cycling. However, the virospheres in macroalgal holobionts remain largely underexplored, representing a critical knowledge gap. Here we unveil that the holobiont of kelp (*Saccharina japonica*) harbors highly specific and unique epiphytic/endophytic viral species, with novelty (99.7% unknown) surpassing even extreme marine habitats (e.g. deep-sea and hadal zones), indicating that macroalgal virospheres, despite being closest to us, are among the least understood. These viruses potentially maintain microbiome equilibrium critical for kelp health via lytic-lysogenic infections and the expression of folate biosynthesis genes. *In-situ* kelp mesocosm cultivation and metagenomic mining revealed that kelp holobiont profoundly reshaped surrounding seawater and sediment virus–prokaryote pairings through changing surrounding environmental conditions and virus–host migrations. Some kelp epiphytic viruses could even infect sediment autochthonous bacteria after deposition. Moreover, the presence of ample viral auxiliary metabolic genes for kelp polysaccharide (e.g. laminarin) degradation underscores the underappreciated viral metabolic influence on macroalgal carbon cycling. This study provides key insights into understanding the previously overlooked ecological significance of viruses within macroalgal holobionts and the macroalgae–prokaryotes–virus tripartite relationship.

## Introduction

Marine macroalgae, commonly known as seaweed, are ecologically and economically vital organisms that form architectural cornerstones of coastal marine ecosystems [1]. With an impressive fixation of 2.9 Pg C·m^−2^·y^−1^, seaweeds drive coastal primary productivity and contribute significantly to global carbon sequestration [2]. Currently, seaweed farming is attracting global attention as a promising way to achieve carbon neutrality [3]. Like land plants, macroalgae engage in intricate symbiotic relationships with associated microbes, including prokaryotes and viruses throughout their life cycle [4]. These microorganisms comprise complex communities living within algal tissues as endophytes, inhabiting the algal surface as epiphytes [1], or residing in the immediate surrounding environment (phycosphere) [5]. Collectively they constitute the seaweed holobiont, which is increasingly recognized to influence host seaweed physiology, growth, stress tolerance, and defense [6–9]. Accumulating evidence indicates that seaweed-associated microbial assemblages are compositionally and functionally distinct from the ambient seawater microbiome [5]. They vary spatio-temporally with changes in seaweed growth stage, health condition, and abiotic factors [10, 11]. However, abnormal shifts in seaweed-associated microbial communities often underpin seaweed disease and deterioration [7, 12]. Hence, a delicate equilibrium exists between macroalgae-associated microbes that dictate host health.

Although the role of host seaweed and abiotic factors in driving the dynamics of the seaweed-associated microbiota has been acknowledged [13], the role of viruses, the most abundant biological entities in the oceans [14], remains an enigma. Viruses are the key biotic mortality drivers of virtually all marine life forms including bacteria and algae [15]. Beyond their conventional role in cell lysis, they also modulate host metabolisms by expressing auxiliary metabolic genes (AMGs) and redirecting the host gene expression, thereby indirectly affecting biogeochemical fluxes within microbiomes and ecosystems [16, 17]. Hence, viruses may likely play a pivotal yet overlooked role in shaping the dynamics of macroalgal holobionts and may regulate macroalgae-microbiota interactions. Through lytic and lysogenic infections of specific microbes, viruses may influence seaweed-associated microbial community dynamics and functions [18]. Additionally, viruses may also directly infect seaweeds as reported previously [19]. However, viral ecology in macroalgal ecosystems remains vastly underexplored compared with prokaryotic communities, representing a critical knowledge gap.

Modern metagenomic technique serves as a powerful tool to explore the hidden viral dynamics directly from environmental samples by enabling the culture-independent reconstruction of viral genomic information [20]. Previous metagenomic surveys have revealed diverse DNA viruses including endophytic or endogenous viral elements (EVEs), primarily affiliated with nucleocytoplasmic large DNA viruses (NCLDVs) [19, 21] and prokaryotic phages associated with the seaweed such as red macroalga *Delisea pulchra* and brown alga *Ecklonia radiata* [22, 23]. Moreover, insights into free-living viral communities of macroalgae and microalgae bloom seawater revealed that viruses may modulate the succession of bacterial communities during and post-bloom phases [18, 24, 25]. Fluctuations in *Phycodnaviridae* and ssRNA viruses have also been linked to the collapse of microalgal blooms [26, 27]. However, no study has yet provided an in-depth characterization of macroalgal virosphere and the factors governing their dynamics. Furthermore, the influence of macroalgae on surrounding seawater and benthic virioplankton dynamics is also unknown.

In this study, we performed an extensive characterization of the virosphere associated with the brown macroalga *Saccharina japonica* (kelp), one of the most important farmed kelp species in the world [2]. We recovered thousands of unique DNA viral genomes and characterized their dynamics within the kelp holobiont (as epiphytic and endophytic viruses) and surrounding kelp phycosphere. By analyzing viral diversity, predicted hosts, lifestyles, phage–host interactions, and AMGs, we provide new insights into the structure and putative roles of the kelp virosphere. Furthermore, by monitoring in-situ environmental factors and prokaryotic community structure, we reveal the key drivers governing kelp virome dynamics. Additionally, we delineate the influence of kelp on surrounding seawater viruses through a simulated kelp cultivation mesocosm experiment, and on benthic viruses within sediments underlying kelp farming areas. Our findings provide new insights into understanding viral forces governing macroalgae microbiomes and the profound shaping effects of the macroalgae on viral communities and functions in surrounding seawater and sediments.

## Materials and methods

### Experimental setup and configuration of the mesocosm experiment

A 63-h mesocosm experiment was conducted in the Sanggou Bay, China (37.145° N 122.555° E, Fig. 2A) in July 2021 using four transparent thermoplastic polyurethane enclosures (covered but air-permeable) with ~565 L of untreated natural seawater. One kelp frond (*S. japonica*, ~2 m long, ~1.1 kg wet weight) per enclosure was suspended in two enclosures (Fig. 2B), whereas remaining two were controls without kelp. All enclosures were secured and cultured under in situ conditions for ~63 h. Abiotic factors including temperature, pH, carbon, nitrogen, phosphorus, and sulfur concentrations were monitored over the course of the experiment along with other biotic factors including prokaryotic and viral abundance (see Supplementary Methods for detailed descriptions).

### Samples collection and preprocessing

At 0 and 63 h, ~55 L seawater was collected per enclosure and passed through 20 μm mesh, to discard large particles. Kelp fronds were also collected and gently rinsed with 0.2 μm filtered sterile seawater (FSW). Kelp surface mucus samples were swabbed using sterile cotton swabs. Mucus-cleaned kelp tissues were rinsed twice with FSW, cut into small pieces using a sterile blade, flash-frozen in liquid nitrogen, and stored at −80°C until further analysis.

Biological replicates of undisturbed sediment cores were collected from three bay locations (S3, S9, and S12, Fig. 2A), beneath the low-density kelp farming area (inner bay), the high-density kelp farming area (mouth of the bay), and the no kelp farming area (adjacent outer sea), respectively, using a Kajak Sediment Sampler (Kc Denmark A/S, Denmark) in June 2020. Cores were sectioned into 3 cm layers and stored at −80°C until further processing.

### DNA extraction and sequencing

For 16S rRNA gene sequencing, 600 ml seawater subsamples were filtered through a 0.2 μm polycarbonate membrane (Millipore, Ireland). Total genomic DNA was extracted using MagPure Soil DNA LQ Kit (Magan, China) from the membranes and sediment, Plant DNA Extraction Mini Kit B (Mabio, China) from kelp tissue, and Advanced Soil DNA Kit (mCHIP BioTech, China) from kelp mucus. The V3-V4 region of the 16S rRNA gene for kelp and seawater samples (barcoded primers: 338F: 5’-GTGCCAGCMGCCGCGG-3′ and 806R: 5’-GGACTACHVGGGTWTCTAAT-3′) and V4 region for sediment samples (barcoded primers: 515F: 5’-GTGCCAGCMGCCGCGG-3′ and 806R: 5’-GGACTACHVGGGTWTCTAAT-3′) were amplified by Polymerase Chain Reaction (PCR). For metagenomic analysis of cell-enriched fraction (> 0.22 μm), 50 L of seawater was filtered through 0.2 μm pore size hydrophilic polyethersulfone membrane (Pall Corp., USA). Total genomic DNA from the membranes and sediment samples was extracted using DNeasy PowerSoil Pro Kit (QIAGEN, Germany), and from the kelp samples using the kits previously utilized for amplicon sequencing.

For viromics (virus-enriched fraction, < 0.22 μm), 50 L seawater samples of the above filtrate (< 0.2 μm) were concentrated to ~1.5 L final volume by tangential flow filtration (Pellicon® 2 Cassette, Biomax® polyethersulfone 30 kDa). The concentrated viral particles were collected by iron chloride flocculation [28, 29] and recovered on 0.8 μm polycarbonate membranes (Millipore, Ireland) by low-pressure filtration (< 15 psi). Total genomic DNA was extracted using TaKaRa MiniBEST Viral RNA/DNA Extraction Kit Ver.5.0 (TaKaRa, China) followed by whole viral genome amplification using the Illustra™ Ready-To-Go GenomiPhi V3 DNA Amplification Kit (GE Healthcare, USA). High-throughput sequencing of the 16S rRNA gene amplicons and extracted DNA samples were performed on the Illumina NovaSeq 6000 platform (Illumina). See Supplementary Methods for further details.

### Metagenome assembly and identification of virus contigs

Raw reads were quality filtered using the KneadData pipeline v0.6.1 (https://github.com/biobakery/kneaddata), to remove low-quality reads (Q < 20) and adapters. For viromes, duplicate reads introduced by DNA amplification were removed using FastUniq v1.1 [30]. Clean reads were assembled separately using metaSPAdes v3.13.0 [31] and co-assembled (per sample type) using Megahit v1.2. 9 [32] to capture low-abundance viruses, with default parameters. All assembled contigs were merged and mapped back to reads using Bowtie2 v2.4.2 [33], and unmapped reads underwent cross-sample assembly with Megahit [32].

Viral contigs were identified from cell-enriched and virus-enriched, kelp (tissue and surface mucus) and sediment metagenomes using VirSorter v2.2.3 (—min-length 5000 —min-score 0.75 —hallmark-required-on-short, —include-groups dsDNAphage, NCLDV, RNA, ssDNA, lavidaviridae) [34], VirFinder v1.1 [35], DeepVirFinder v1.0 [36], CAT v5.1.2 [37], and the JGI Pipeline (Table S13) [38]. In detail, contigs >5 kb were piped through VirSorter2, VirFinder, and DeepVirFinder, and those contigs predicted with VirSorter score ≥ 0.75 or VirFinder score ≥ 0.7 (*P* < .05) or DeepVirFinder score ≥ 0.7 (*P* < .05) were retained. Among these contigs, those that met one of the following criteria were considered as viral: (i) VirSorter score ≥ 0.9; (ii) VirFinder score ≥ 0.9 and *P* < .05; (iii) DeepVirFinder score ≥ 0.9 and *P* < .05; (iv) VirSorter score ≥ 0.75 AND VirFinder score ≥ 0.7 (*P* < .05) AND DeepVirFinder score ≥ 0.7 (*P* < .05). The remaining contigs that did not pass above criteria were imported into CAT and considered as viral if <40% of the contig length was annotated as prokaryotic or eukaryotic (gene length relative to contig length). Additionally, the contigs that failed to pass through VirSorter2, VirFinder, and DeepVirFinder were retrieved to identify viral contigs using the default three filters of the JGI Pipeline. Finally, all identified viral contigs were merged, and deduplicated using CD-HIT v4.8.1 (cd-hit-est; −c 0.99) [39]. The nonviral regions were removed using CheckV v0.7.0 [40]. Viral operational taxonomic units (vOTUs) were defined by clustering nonredundant contigs at 95% average nucleotide identity >85% alignment fraction [20] using the CheckV genome clustering script (https://bitbucket.org/berkeleylab/checkv). The quality of vOTUs was estimated using CheckV. Viral binning was conducted via the PHAMB pipeline [41].

For further details on the virus and prokaryotic community analysis see Supplementary Methods.

### Abundance profiles of viruses and prokaryotes in metagenomes

Relative abundances of viruses and prokaryotes were quantified as reads per kilobase per million mapped reads (RPKM) metric [42, 43]. In brief, the cleaned reads from each sample were mapped to viral contigs, vMAGs, and pMAGs using BamM v1.7.3 (https://github.com/Ecogenomics/BamM) at ≥95% identity over ≥75% coverage. Coverage profiles of vOTUs (“contig” mode) and MAGs (“genome” mode, including vMAGs and pMAGs) were generated using CoverM v0.6.1 (https://github.com/wwood/CoverM) with —min-read-percent-identity-pair 95, —min-read-aligned-percent-pair 75, —methods rpkm, —proper-pairs-only, and —min-covered-fraction 75 for (vOTUs), 20 (vMAGs), or 10 (pMAGs) [43–45].

To detect the presence of vOTUs_k_ in publicly available macroalgal metagenomes, the metagenomes of brown, red, and green macroalgae (accession no. = PRJEB50838, ENA database) [46] and kelp viromes (accession no. = SRX3446198–203, NCBI SRA database) [23] were retrieved and processed as described above. The presence of a vOTU was inferred at RPKM >0.

### Differential abundance of viruses and prokaryotes in the mesocosm experiment

Differential analysis was employed to identify viruses and prokaryotes exhibiting significant differences following the 63-h mesocosm experiment. Raw counts from the DADA2 (ASVs, see Supplementary Methods) and CoverM (vOTUs, vMAGs, and pMAGs; with “—methods count”) were used as input into DESeq2 v1.26.0 [47]. The viruses and prokaryotes that were significantly (adjusted *P* < .05) enriched (log_2_ fold change >1) or reduced (log_2_ fold change <1) in kelp mesocosms were retrieved. The significantly differed sequences in no-kelp mesocosms (control) were discarded, to account for confounding factors.

### Identification of shared vOTUs and vMAGs between different sample types

Shared vOTUs or vMAGs between different sample types were determined by transforming relative abundance tables into binary presence-absence matrices, where a relative abundance of >0 was used as a threshold to determine the presence of a vOTU or vMAG. The proportion of shared vOTUs of paired samples was calculated using the reported equation and code [48].

Shared viral species between different sample types as well as with the IMG/VR4.0 [49] and NCBI RefSeq Virus (release 210) databases were determined by clustering vOTUs and vMAGs using the CheckV genome clustering method provided by CheckV (https://bitbucket.org/berkeleylab/checkv) as described above.

Shared viral genera (or subfamilies) were determined by constructing a gene-sharing network by vConTACT2 (—rel-mode 'Diamond'; —pcs-mode MCL; —vcs-mode ClusterONE), using the predicted protein sequences of vOTUs and vMAGs with sufficient genetic information (with a size of >10 kb or completeness of >90%) [50] as input. The gene-sharing network was visualized in Gephi v0.9.2 [51] with the Fruchterman Reingold layout.

### Virus-host linkage analysis

Three *in silico* approaches were used to match vOTUs and vMAGs to pMAGs (Table S5), linking each virus to putative “*in-situ*” hosts: (i) *CRISPR matching*- CRISPR spacers of all pMAGs predicted by metaCRT [52, 53] were aligned with the vOTUs and vMAGs using BLASTn to identify the viral protospacers. Hits with ≥95% identity and 100% coverage and ≤ 1 mismatch were considered as the putative virus-host pairs [42, 52]. (ii) *tRNA matching-* tRNA genes in pMAGs and vOTUs (and vMAGs) were predicted using tRNA-scan v2.0.7 [54] and compared using BLASTn at 100% coverage and identity [52]. (iii) *Direct genomic alignment*- vOTUs were directly aligned with pMAGs using BLASTn and matches with the thresholds of bitscore >50, E-value <10^−3^, identity >70%, length ≥ 2500 bp, coverage relative to vOTUs >75%, and coverage relative to pMAG contigs <66.7% were considered the most confident host predictions [52, 55]. This approach was not applied in matching vMAGs and pMAGs, because of the uncertainty of sequence order and the presence of gaps in vMAGs.

iPHoP was employed to predict the “*ex-situ*” hosts of viruses using the proposed cutoff of confidence score > 90 [56]. Besides, tRNA sequences in vOTUs and vMAGs were compared against the public tRNA sequences that were also predicted by tRNA-scan and deposited in the GtRNAdb database (release 19, http://gtrnadb.ucsc.edu/) as described above.

Due to the lack of current tools for predicting hosts of eukaryotic viruses (like NCLDVs) [55], we utilized genomic context analysis to tentatively infer the putative relationships between eukaryotic viruses and hosts, following previous studies [57]. NCLDV genes were taxonomically annotated by BLASTP alignment (E-value of 1e-5, identity of ≥30%, and query coverage of ≥50%) to the NCBI NR database (released December 2021). The top hit was retained.

### Viral functional profiles and identification of AMGs.

AMGs were identified from vOTUs and vMAGs using DRAM-v v1.2.0 [58] with default parameters and minor modifications [42, 50, 59]. Viral genes with a confidence score of 1–3 and metabolic flag (“M”) were selected as the high-confidence AMGs [58]. To be conservative, those AMGs without specific gene descriptions or related to glycosyltransferases, ribosomal proteins, organic nitrogen metabolism, and nucleotide metabolism were not considered [42, 59] as were AMGs near assembly gaps in vMAGs. The functional activity and phylogenetic status of AMGs were bioinformatically verified, see Supplementary Methods for details. Genome maps and alignments for AMG-carrying vOTUs and vMAGs were visualized using Easyfig v2.2.5 [60].

### Prophage identification from kelp-associated prokaryotic genomes

We collected 2584 prokaryotic genomes from a previous study [46], including those from epiphytic bacteria of red macroalgae (*Gelidium* sp. and *Grateloupia* sp., *n* = 1148), brown macroalgae (*Saccharina* sp., *n* = 151), green macroalgae (*Ulva* sp., *n* = 502), seawater (*n* = 469), and sediment (*n* = 314). Also, prokaryotic metagenome-assembled genomes recovered from this study were included, including those from kelp epiphytic or endophytic bacteria (*n* = 95) and prokaryotes in seawater (*n* = 498) and sediment (*n* = 257). Prophages were identified from these genomes using geNomad v1.7.0 [61] (virus_score > 0.7). Functional annotations (including AMG identification) for these prophages were performed by VIBRANT v1.2.1 [62] (default setting).

### Statistical analysis

All analyses were performed in R v3.6.3 unless otherwise stated. The linear regression analysis was performed using Pearson’s correlation using the *ggcorrplot* and *ggpubr* packages. Pairwise Spearman’s correlation analysis and the Mantel test, showing the relationships between environmental parameters and the microbial community were performed using the *linkET* v0.0.7 (https://github.com/Hy4m/linkET) package based on calculated microbial (Bray–Curtis) and environmental (Euclidean) distances. The correlation between dominant bacterial genera and environmental factors was evaluated by Spearman’s analysis using the *psych* package. *Vegan*, *ape*, and *amplicon* packages were used to estimate the alpha diversity indexes (Chao1 and Shannon) and perform principal coordinate analysis (PCoA) and analysis of variance using distance matrices (ADONIS) analyses. Data visualization and statistical tests were performed by Chiplot (https://www.chiplot.online/), ImageGP (http://www.bic.ac.cn/BIC/#/), and R using ANOVA, Tukey’s multiple comparisons tests, *T*-tests, and non-parametric Wilcoxon rank sum tests.

## Results

### Highly unique viruses inhabit kelp and may contribute to microbial equilibrium in kelp holobiont and kelp growth

**Figure 1:**
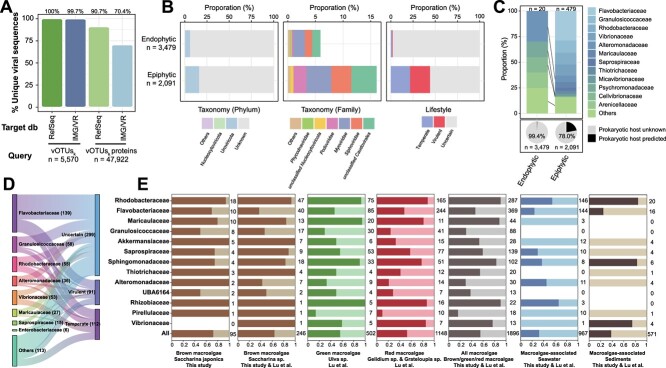
Profiles of kelp endophytic and epiphytic viruses. (A) The proportions of unique vOTUs_k_ and viral proteins predicted from kelp endophytic and epiphytic viral communities (vOTUs_k_) in comparison to IMG/VR and NCBI Refseq virus databases. (B) Community (phylum and family level) and lifestyle compositions of kelp endophytic and epiphytic viruses (vOTUs_k_). (C) The proportion of kelp endophytic and epiphytic viruses (vOTUs_k_) with predicted prokaryotic hosts and their host compositions. (D) Sanky plot showing the lifestyles of kelp endophytic and epiphytic phages (vOTUs_k_ and vMAGs_k_) infecting the main bacteria groups dominated in kelp (left). (E) The proportions (indicated by dark color) of prophage-carrying bacterial genomes (including draft genomes of cultivated bacteria and metagenome-assembled genomes) among different bacteria groups dominated in kelp. Bacterial genomes were retrieved from this study and a previous study [46].

A total of 5570 viral operational taxonomic units (vOTUs, non-redundant viral contigs >5 kb, labeled as vOTUs_k_) and 83 non-redundant viral metagenome-assembled genomes (vMAGs, labeled as vMAGs_k_) were identified from kelp tissue and kelp surface mucus metagenomes (Tables S1 and S2; Supplementary Text). Among these vOTUs_k_, > 99% of them and > 70% of viral genes were unique compared to the IMG/VR v4.0 and NCBI RefSeq Virus databases (Fig. 1A). Furthermore, these viruses inhabiting macroalgae appear to exhibit specificity, as only 2.1%, 0.6%, and 0.1% of all vOTUs_k_ identified here were detected in the public metagenomes of *Saccharina* sp. (brown macroalgae), *Ulva* sp. (green macroalgae), and *Grateloupia* sp. (red macroalgae), respectively [46]. Among 5570 vOTUs_k_, 3479 were from the kelp tissues and 2091 from surface mucus and were tentatively recognized as potential endophytic and epiphytic viruses, respectively (Table S1). Among these, 799 vOTUs_k_ were found to be shared between the kelp tissues and surface mucus samples (Table S1). In comparison to epiphytic viruses, endophytic viruses contained a higher proportion of viral “dark matter” with unknown taxonomy (94.1% vs 83.9%), lifestyle (i.e. lytic or lysogenic infections, 98.0% vs 55.9%), and predicted prokaryotic hosts (99.4% vs 78.0%) (Fig. 1B and C).

**Figure 2:**
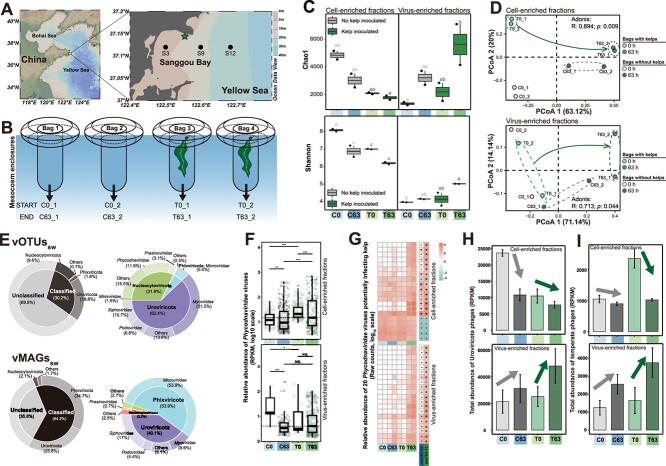
Overview of the kelp mesocosm experiment and the changes in virioplankton communities. (A) Map of the Sanggou Bay showing sampling sites where the mesocosm experiment was performed (pentagram) and the sediment samples were collected (dots). (B) Schematic plot showing the setup and configuration of the kelp mesocosm experiment. Changes in alpha and beta diversity of virioplankton communities in different fractions, reflected by (C) Chao1 and Shannon indexes and (D) PCoA. In (C), different letters indicate significantly different groups (*P* < .05, ANOVA, Tukey HSD). (E) Community compositions (phylum and family level) of virioplankton represented by vOTUs_sw_ and vMAGs_sw_. Changes in the relative abundance of (F) *Phycodnaviridae* viruses and (G) those potentially infecting kelp. In (F) and (G), *P < .05 indicates significant differences based on the Wilcox test and DEseq2 analysis, respectively. Changes in the total relative abundance of (H) Uroviricota phages and (I) temperate phages. Control and kelp-inoculated groups are denoted as “C” and “T” respectively.

Both epiphytic and endophytic viruses inhabiting kelp contained eukaryotic viruses from the Nucleocytoviricota phylum (i.e. nucleocytoplasmic large DNA viruses, NCLDVs) (Fig. 1B). Based on genomic context taxonomic analysis, we found two endophytic *Phycodnaviridae* viruses likely infecting kelp, as they contained multiple Laminariaceae-homologous genes (Table S3). In contrast, the epiphytic eukaryotic viruses, represented by eight vOTUs_k_ and one vMAGs_k_, are predicted to include viruses infecting protistan hosts, like amoeboid protozoa, fungi, as well as microalgae (Table S4). Furthermore, the majority of taxonomically assigned epiphytic and endophytic viruses belonged to bacteriophage families *Myoviridae*, *Siphoviridae*, and *Podoviridae* of the *Caudovirales* order (recently renamed as the class Caudoviricetes), with a higher proportion in the epiphytic fraction compared to the endophytic one (Fig. 1B). The predicted hosts for the epiphytic bacteriophages were mainly epiphytic bacteria or the dominant bacteria in the phycosphere, including members of the *Flavobacteriaceae* (*Algibacter*, *Tenacibaculum*, *Polaribacter*, *Aquimarina*), *Granulosicoccaceae* (*Granulosicoccus*), *Rhodobacteraceae*, and *Vibrionaceae* families (Fig. 1C & Supplementary Text). In contrast, fewer predicted prokaryotic hosts of endophytic phages were primarily endophytic bacteria (e.g. Enterobacterales bacteria) (Table S6 & Supplementary Text). At the prokaryotic genus level, the most commonly predicted viral hosts were *Granulosicoccus* and *Vibrio* bacteria, which represent the dominant seaweed beneficial bacteria [9] and opportunistic pathogenic bacteria [6], respectively. Typically, two phages with high-quality genomes (completeness >90%, K-S3C66802, and K-k141_1663699) were predicted to infect *Granulosicoccus* and *Vibrionaceae* bacteria, respectively (Table S6). These two phages were predicted to be lytic (Table S2), indicating their roles in modulating kelp bacterial communities. Additionally, we found that lysogeny might also be a significant lifestyle for phages inhabiting kelp, especially for those potentially infecting core bacterial members of the kelp. For example, predicted phages of *Rhodobacteraceae*, *Granulosicoccaceae*, and *Maricaulaceae* bacteria contained more lysogenic proportion (except for the phages with “uncertain” lifestyle) (Fig. 1D). Moreover, >72% (69 out of 95) of metagenome-assembled genomes (MAG) assembled from kelp (pMAGs_k_) contained prophage regions (Fig. 1E). Concordant with this, analysis of 1801 pMAGs and draft genomes of macroalgae epiphytic bacteria retrieved from a recent study [46] revealed high prophage carriage in macroalgae common core bacteria groups like *Rhodobacteraceae*, *Maricaulaceae*, and *Sphingomonadaceae* (Fig. 1E and Table S7). Besides, the brown macroalgae (*Saccharina* sp.) harbored a higher proportion of prophage-carrying bacteria (~64.2%), compared with green macroalgae (*Ulva* sp., ~56.8%) and red macroalgae (*Gelidium* sp. and *Grateloupia* sp., ~53.1%) (Table S7). Additionally, nearly 20% of the predicted AMGs identified from these prophages were related to the metabolism of cofactors and vitamins, especially folate biosynthesis (Fig. S1), indicating that these prophages might help their bacterial hosts to biosynthesize vitamin or related precursors for macroalgae growth [8].

### Kelp altered seawater virioplankton community with enriched *Phycodnaviridae* viruses and bacteriophages

During the 63-hour kelp cultivation mesocosm experiment (Fig. 2A and B), we observed a substantial increase in virioplankton abundance compared with the controls without kelp (Fig. S2A), indicating that kelp influences surrounding seawater virioplankton communities. We analyzed virioplankton communities in seawater samples from two size fractions: cell-enriched 0.22 ~ 20-μm-size fractions (CFs) and virus-enriched <0.22-μm-size fractions (VFs) at the start and end of the mesocosm experiment. As a result, 12 518 vOTUs (labeled as vOTUs_sw_) and 632 vMAGs (labeled as vMAGs_sw_) were identified (Tables S1 and S2; Supplementary Text). At the end of the experiment, we observed significant (*P* = .009) alterations in the assembly of both CF and VF viral communities in the seawater with kelps from those in the seawater without kelps, as evidenced by the Bray–Curtis dissimilarity PCoA and permutational multivariate ADONIS analysis (Fig. 2D). While comparable alterations in alpha diversity (Chao1 and Shannon) were noticed in control and kelp-inoculated groups after 63 h (Fig. 2C), such significant dissimilarities in beta diversity (Fig. 2D) pointed to distinctive viral community structure in kelp inoculated treatments. In detail, among the notable fraction of vOTUs_sw_ (9.6%) and vMAGs_sw_ (2.1%) comprised of eukaryotic viruses originating from the Nucleocytoviricota phylum (Fig. 2E), *Phycodnaviridae* viruses were enriched with kelps (Fig. 2F), whereas other unclassified Nucleocytoviricota viruses were diminished (Fig. S3A). Among these, 15.7% (70 out of 447) of *Phycodnaviridae* viruses harbored genes homologous to *Laminariaceae*, implying their potential to infect kelp (Table S3). Additionally, bacteriophages (vOTUs_sw_ and vMAGs_sw_) belonging to the Uroviricota phylum were largely enriched in seawater VFs with kelps (Fig. 2H). Among these bacteriophages, temperate phages were increased substantially in the seawater CFs after the introduction of kelp and were substantially reduced after 63-h kelp cultivation, while also largely enriched in seawater VFs (Fig. 2I), suggesting their potential lytic induction and releasing of progeny phages from virocells. Moreover, almost all virus-enriched fractions from seawater exhibited a higher relative abundance of *Microviridae* (ssDNA) viruses (Table S1), likely due to the sequencing biases introduced by multiple displacement amplification which tend to overrepresent small circular ssDNA viruses [63].

Differential analysis at the vOTUs (species) level revealed substantial impacts of kelp cultivation on virioplankton communities, as evidenced by the higher number of affected vOTUs in kelp mesocosms compared to controls (Fig. S3B). Upon excluding vOTUs with the same significant variations in controls, 1748 and 1040 vOTUs were found to be significantly enriched and reduced in kelp mesocosms (Table S8), respectively, representing the sensitive viral species in response to kelp cultivation. Except for the taxonomically unclassified viruses, these vOTUs primarily belonged to the *Caudovirales* order (~57.0%, i.e. bacteriophages) and Nucleocytoviricota phylum (~29.0%, primarily *Phycodnaviridae* family) (Fig. S3C). The majority of these *Phycodnaviridae* viruses (~81.9%) and bacteriophages (~63.8%) were significantly enriched in response to kelp cultivation (Table S8). Among these, 20 significantly enriched *Phycodnaviridae* viruses in seawater CFs and/or VFs containing kelps (Fig. 2g) had the potential to infect kelp. These results indicate that kelp cultivation enriched the potential kelp viruses and bacteriophages in seawater.

### Kelp strengthens virus-prokaryote interaction through prokaryotic community shifts driven by environmental changes

**Figure 3:**
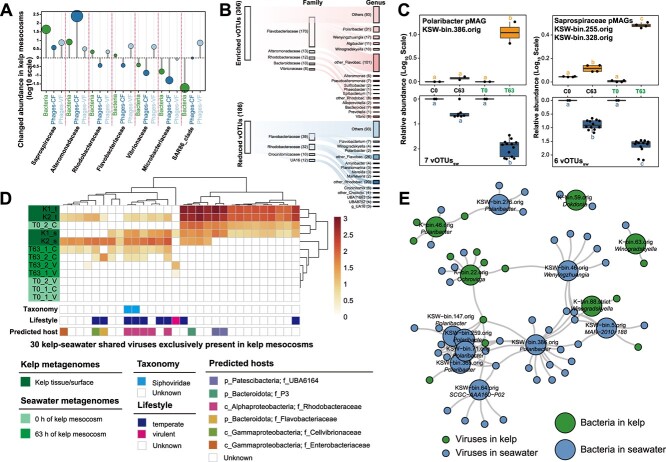
Dynamics of phage-bacterium interactions in the kelp mesocosm experiment. (A) Changes in the relative abundance of bacteria and their predicted phages among different bacterial families in kelp mesocosms. (B) Sanky plot showing the predicted phage-bacteria associations among the significantly differentially abundant phages (vOTUs, left) in response to kelp cultivation. The middle and right bars represent the number of pairings in each bacterial family and genus, respectively. (C) Changes in the relative abundance of a *Polaribacter* bacterium and two *Saprospiraceae* bacteria and their predicted phages in cell-enriched fractions. Different letters indicate significantly different groups (*P* < .05, ANOVA, Tukey HSD). (D) Heatmap showing the kelp-seawater shared viruses exclusively present in kelp mesocosms. Control and kelp-inoculated groups are denoted as “C” and “T” respectively. (E) Interaction network of *Flavobacteriaceae* bacteria (large circles) and phages (small circles) identified from kelp and seawater.

Kelp also contributed to the significant elevation in absolute prokaryotic abundance (Fig. S2A) and significantly promoted virus-prokaryote interactions (Pearson’s *R* = 0.81; *P* < .01, Fig. S2B). Given that viral reproduction relies on susceptible hosts, the prokaryotic communities, significantly influenced by kelp cultivation (Supplementary Text), represent a crucial biotic driver shaping virioplankton communities. We found that the enrichment of some bacterial taxa was accompanied by the enrichment of their corresponding viruses in kelp mesocosms, for instance, *Saprospiraceae* and *Alteromonadaceae* bacteria and their phages (Fig. 3A). Besides, among the significantly enriched vOTUs_sw_ with predicted prokaryotic hosts, ~62.9% (39 out of 62) could potentially infect *Flavobacteriaceae* (mainly *Polaribacter*), *Saprospiraceae*, and *Alteromonadaceae* bacteria (Fig. 3B), which were also significantly enriched in response to kelp cultivation (Fig. S4H). Among these significantly enriched virus-host pairs, seven vOTUs_sw_ potentially infecting *Polaribacter* bacterium (KSW-bin.386.orig) and six vOTUs_sw_ potentially infecting two *Saprospiraceae* bacteria (KSW-bin.255.orig and KSW-bin.328.orig) exhibited a lysogenic lifestyle based on host prediction as evidenced by their potential historical integration into host genomes (reflected by their genomic alignment) (Table S6) and their consistent coexistence with hosts in the seawater CFs (Fig. 3C). Although these virus-host pairs were both significantly enriched in the cell-enriched fractions (*P* < .05, Fig. 3C), *Polaribacter* phages appeared in the virus-enriched fractions only after 63-h kelp cultivation (Table S1), implying their lytic induction and release of progeny phages as mentioned above. Besides lysogenic phages, lytic phages were also enriched in response to kelp cultivation, and nearly one-third of significantly enriched vOTUs_sw_ were virulent phages (Table S8). Although very few significantly enriched lytic phages were linked to their potential in situ prokaryotic hosts, their enrichment was largely attributed to their host enrichment. Thus, both lytic and lysogenic infection, alongside host enrichment, appear to drive kelp-associated virus communities.

In addition to biotic factors, abiotic factors such as environmental changes were also found to affect virioplankton communities in kelp mesocosms. Typically, dissolved organic carbon (DOC), particulate organic carbon (POC), and dissolved oxygen (DO) concentrations changed significantly in kelp mesocosm seawater compared to controls without kelps (Table S9 and Fig. S2A). These environmental changes were significantly correlated with viral abundance and communities through correlation analysis and the Mantel test, respectively (Fig. S2C and D). However, when accounted for the controlling effects of the prokaryotic communities (partial Mantel test), DO, DOC, and POC showed reduced and no correlations with VF and CF viral communities, respectively (Table S10). Distance-based redundancy analysis (db-RDA) further indicated that DO, DOC, and POC did not directly affect viral communities significantly but explained significant prokaryotic community variation (57.3%, 27.4%, and 17.7%, respectively; *P* < .05, Table S10). Together, these results suggested that DO, DOC, and POC primarily shape virioplankton communities by modulating prokaryotic communities in seawater.

### Shared viruses and virus-bacteria connections between kelp and seawater also contribute to the changes in virioplankton communities

Although distinct (Fig. S5B-E), viral communities in kelp and seawater showed some interconnections. Specifically, we identified 34 vOTUs and 6 vMAGs that were simultaneously present in both kelp and seawater metagenomes (Fig. S6). Among these, 30 viruses were exclusively found in kelp mesocosm seawater (Fig. 3D), indicating the potential virus migration between kelp and surrounding seawater. These shared viruses were predominantly kelp epiphytes or present in seawater CFs and mostly predicted as temperate phages infecting *Rhodobacteraceae* bacteria (Fig. 3D), suggesting kelp may introduce epiphytic *Rhodobacteraceae* lysogens into surrounding seawater (Fig. 1E). Moreover, two viral species and 17 viral genera/subfamily were shared between kelp and seawater metagenomes (Fig. S5C), both suggesting the interconnections of viruses in kelp and seawater.

We examined the extent of virus–host interactions and found viral cross-infections to bacteria between kelp and seawater environments (Fig. 3E). We observed that 13 kelp epiphytic and 1 endophytic viruses (vOTUs_k_) were linked to 33 different bacteria in seawater (pMAGs_sw_), whereas 23 virioplanktons (22 vOTUs_sw_ and 1 vMAGs_sw_) were linked with 17 different kelp epiphytic bacteria (pMAGs_k_) (Table S6). A bacterioplankton (KSW-bin.386.orig) belonging to the *Polaribacter* genus, which was significantly enriched in response to kelp cultivation, was predicted to be infected by 16 viruses from seawater (including 7 significantly enriched vOTUs_sw_) and 2 viruses from kelp (vOTUs_k_) (Fig. 3e & Table S6). Additionally, a kelp epiphytic bacterium (K-bin.22.orig) belonging to the *Ochrovirga* genus (*Flavobacteriaceae*) was predicted to be infected by five viruses from kelp (vOTUs_k_) and four viruses from seawater (all were significantly enriched vOTUs_sw_) (Fig. 3E and Table S6). Thus, the introduction of kelp epiphytic bacteria and viruses into the surrounding seawater also likely influences virioplankton communities.

### Kelp farming contributes to the unique viral communities in the seafloor surface sediments and the burial of considerable kelp epiphytic viruses into sediments

**Figure 4:**
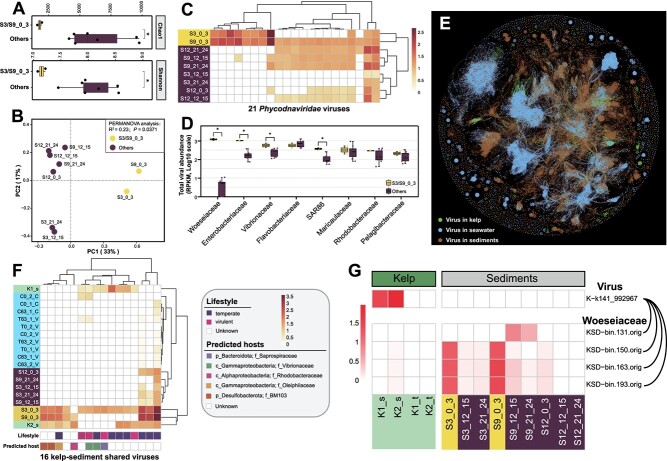
Influence of kelp farming on benthic viral communities and virus-host interactions. Alpha and beta diversity analyses showing the endemic viriobenthos communities in surface sediments beneath kelp-farming areas, reflected by (A) Chao1 and Shannon indexes and (B) PCoA. (C) Heatmap showing the relative abundance of the enriched 22 *Phycodnaviridae* viruses in surface sediments beneath kelp-farming areas. (d) Relative abundance of viruses potentially infecting different bacteria families in sediments. (E) Gene-sharing network of viruses from kelp, seawater, and sediments. The edges indicate protein cluster similarity. (F) Heatmap showing the relative abundance, predicted lifestyle, and hosts of 16 viruses shared between kelp and sediments. (G) Heatmap showing the relative abundance of a kelp epiphytic phage and their predicted *Woeseiaceae* hosts in benthic sediments.

In total 21 291 vOTUs (labeled as vOTUs_sd_) and 369 vMAGs (labeled as vMAGs_sd_) were identified from bulk metagenomes of sediments at different depths and kelp farming areas (Tables S1 and S2; Supplementary Text). Kelp farming significantly shaped viral communities in surface sediments of the kelp farming areas, evidenced by their lower richness and evenness (Fig. 4A), distinct community composition (Fig. 4B), and high percentage of unique viruses (Fig. S5E) compared to other sediments. Among the potential eukaryotic Nucleocytoviricota viruses (~1.1% of vOTUs_sd_ and ~ 0.8% of vMAGs_sd_), 23 harbored genes homologous to *Laminariaceae* (Table S3) and 83 were potential *Phycodnaviridae* viruses (Tables S1 and S2), and 21 of them were enriched in surface sediments of the kelp farming area (Fig. 4C), suggesting the potential enrichment of viruses infecting kelp because of the kelp farming. Furthermore, among the potential bacteriophages with predicted prokaryotic hosts (~9.2% of vOTUs_sd_), those infecting Gammaproteobacteria (*Woeseiaceae*, *Enterobacteriaceae*, *Vibrionaceae*, and *SAR86*) were significantly enriched in the surface sediments of the kelp farming areas (Fig. 4D).

A substantial portion of viruses was found to be shared between seawater and sediments (Fig. S5B, C and E), as also evidenced by the phylogenetic close clustering of seawater and sediment viruses primarily from NCLDVs and (previously designated) Caudovirales groups (Fig. S7). Intriguingly, benthic viruses also displayed connections with kelp-associated viruses. For instance, PCoA analysis revealed that viral communities in the surface sediments of the kelp-farming areas were more similar to viral communities inhabiting kelp than those in non-farming sediments (Fig. S5D). Phylogenetic analyses indicated that NCLDVs in kelp primarily clustered with those from sediments, and many head-tailed phages in kelp were also grouped with phages from sediments (Fig. S7). Additionally, the gene-sharing network (Fig. 4E) also illustrated that viruses in kelp tended to be clustered with viruses in sediments (forming 23 viral clusters) rather than viruses in seawater (forming 17 viral clusters, Fig. S5C). Between kelp and sediment metagenomes, 16 viruses were shared, among which 4 viruses were also detected in seawater, whereas the other 12 viruses were exclusively detected in kelp and sediments (Fig. 4F). This suggests two potential viral transport pathways from kelp: (i) direct deposition of viruses from kelp into seafloor sediments or (ii) indirect transport involving release into seawater followed by subsequent deposition in sediments. Additionally, these kelp-sediment shared/clustered viruses were all kelp epiphytic viruses, and these kelp epiphytic viruses transported to sediments may impact autochthonous bacteria. For instance, a kelp epiphytic virus (K-k141_992967) was predicted to infect four different *Woeseiaceae* (Gammaproteobacteria) bacteria (KSD-bin.131.orig, KSD-bin.150.orig, KSD-bin.163.orig, and KSD-bin.193.orig), which were enriched in the surface sediments of the kelp farming areas (Fig. 4G) and are considered as autochthonous bacteria in the global marine sediments [64].

### Kelp-associated phages carry laminarinase genes (AMGs) that may assist host *Flavobacteriaceae* in degrading kelp laminarin

**Figure 5:**
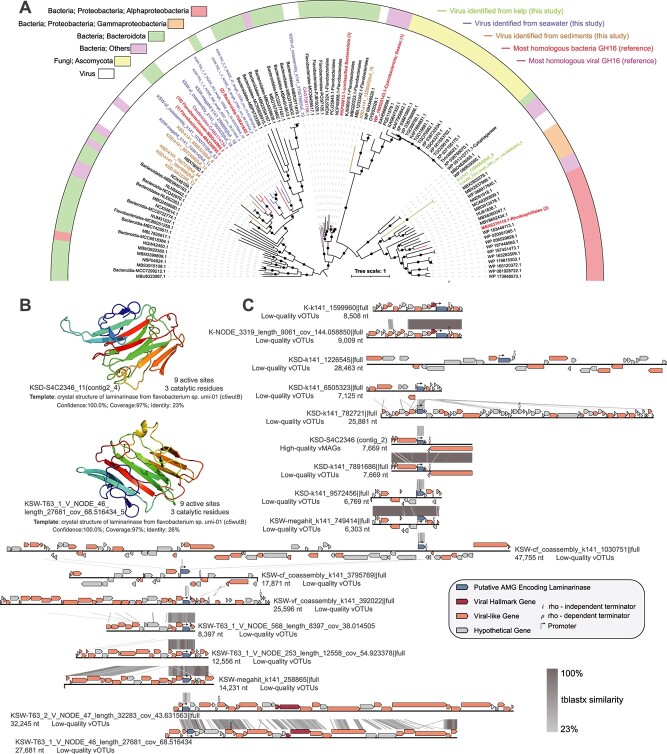
Genomic context, predicted protein structure, and phylogeny of the viral laminarinase genes. (A) Maximum likelihood tree of the 17 putative viral laminarinases from kelp-associated viruses, and 88 bacterial or viral laminarinase sequences from the NCBI NR database. The proportional circles represent internal nodes and bootstraps. (B) Predicted tertiary structures of two virally expressed AMGs. (C) Genome maps of the vOTUs or vMAGs showing the location of viral laminarinase genes.

A total of 26, 231, and 197 high-confidence viral AMGs were identified from viral genomes (including vOTUs and vMAGs) of kelp, seawater, and sediments, respectively (Table S11). All of these AMGs were verified to be flanked by viral hallmark genes. Among these, a higher number of viral AMGs were related to the glycoside hydrolase (GH) family, including 17 GH16 (PF00722.23) laminarinases (Table S12) from seawater (9), kelp (2), and sediments (6) viruses. The majority of the viruses that carried these laminarinase AMGs were enriched in the seawater with kelp cultivation (at the end of the mesocosm experiment), kelp surface, and sediments of the kelp farming areas (Fig. S8). Additionally, these AMGs likely encoded functionally active laminarinases with promoters and terminators predicted upstream and downstream of these genes (Fig. 5C). At the amino acid sequence level, the protein products of 11 out of 17 AMGs contained conserved functional domains and multiple enzymatically active sites and catalytic residues characteristic of the laminarinase family (Table S12). *In silico*, 13 out of 17 AMGs had high-confidence predicted protein structures matching laminarinases crystal structure with 100% confidence and > 90% alignment coverage (Fig. 5B and Table S12). Moreover, phylogenetic analysis showed that these viral laminarinases were primarily clustered with those from Bacteroidetes (*Flavobacteriaceae*) (Fig. 5A), indicating that the viruses carrying these AMGs possibly infected these bacteria and that these AMGs were likely horizontally transferred from the host genomes during the viral infection. Besides, the viral laminarinases identified in this study formed an independent new evolutionary cluster compared to the laminarinases sequences deposited in NCBI (Fig. S9). This highlights the uniqueness of the laminarinases encoded by kelp-associated viruses.

## Discussion

Similar to terrestrial plants, marine macroalgae harbor unique prokaryotic communities within their holobionts or phycospheres, significantly influencing the health and resilience of the host macroalgae [7–9]. Yet, the understanding of macroalgae-associated viruses, the most abundant microorganisms in the ocean [4], remains limited. Understanding the dynamics of associated viruses is crucial for comprehending the microbiome dynamics and ecological health of both macroalgae and marine environments.

This study provides an in-depth characterization of viruses associated with the ecologically and economically important macroalgae kelp (i.e. *Saccharina japonica*) widely farmed in Asia [65]. We uncovered an extremely diverse viral community inhabiting kelp as epiphytes and endophytes, encompassing over five thousand distinct viral species (Table S1). These viruses were highly unique with >99% of viral species unrelated to previously known viral species. The uniqueness of these viruses surpassed even those found in extreme marine habitats such as deep-sea (with the proportion of unique viral sequences of 30.0–49.2%) [66], hydrothermal vents (66.4%) [67], cold seeps (70.1%) [42], and hadal trenches (96.9–97.7%) [52, 59, 68] (Fig. S10). To our knowledge, only one prior study has explored the DNA virus community inhabiting kelp, featuring primarily bacteriophages and *Phycodnaviridae* viruses [23], which aligns with our findings (Fig. 1B). However, no viral sequences identified from our study can be detected from this previously reported kelp virome [23], and very few viruses (<2.1%) were shared with other macroalgae metagenomes [46], suggesting the distinct DNA viral communities among different macroalgae individuals. Additionally, among the viruses inhabiting kelp, endophytic viruses exhibited greater novelty than their epiphytic counterparts (Fig. 1B and C), emphasizing the underexplored realm of endogenous viral elements (EVEs) in brown algae. This is particularly intriguing considering that EVEs have been widely identified in green algae and can influence their host genomes [69]. In summary, macroalgae, particularly in the context of endophytic viruses, represent an untapped reservoir housing an extensive diversity of enigmatic viral elements that warrant thorough investigation in future studies.

It’s logical to assume that kelp endophytic NCLDVs likely target kelps as their primary eukaryotic hosts. This could be attributed to the fact that kelp viruses, specifically those of the *Phaeovirus* genus within the *Phycodnaviridae* family, typically undergo a lysogenic phase in their life cycle [21, 70]. Given the uncertainty surrounding the lifestyle of these endophytic *Phycodnaviridae*-like members and the lack of observable morphological symptoms of infection on the growing kelp sporophytes, it remains unclear whether these viruses represent actively infectious agents or inactivated viral elements. Conversely, kelp epiphytic NCLDVs are more inclined to infect amoeboid protozoa, fungi, or microalgae. This preference may arise from the prevalence of these microorganisms on the kelp surface [71, 72]. Beyond NCLDVs, bacteriophages were abundant in kelp virome, mostly because kelp-surfaced biofilms are hotbeds of bacterial activity. Here, we observed many virulent phages targeting both previously reported kelp opportunistic pathogenic bacteria, like *Pseudoalteromonas*, *Vibrio*, *Colwellia*, and others [6, 7, 12], and kelp-beneficial bacteria, like *Granulosicoccus* sp. (K-bin.9.strict) [9]. The former is like “time bombs” that can rapidly turn into pathogens following microbiome dysbiosis due to unfavorable environmental conditions [7], adversely impacting kelp growth. The latter, despite their benefit for kelp growth, may also lead to kelp microbiome dysbiosis following their unchecked proliferation, negatively affecting kelp health. Thus, the virulent phages in kelp endophytic/epiphytic viral communities play a crucial role in suppressing excessive bacterial growth. However, we also found lysogenic infections were more widespread among brown macroalgae symbiotic bacteria with >60% carrying prophages, compared to prokaryotes inhabiting macroalgae-associated seawater and sediments (< 40%, Fig. 1e), as well as the global marine prokaryotes (~40%) [73]. This finding contrasts with the traditional view that lysogeny is primarily prevalent in oligotrophic environments [17, 73], as kelp surface habitats are rich in nutrients, oxygen, and microbial activity [1, 71]. We speculate that the prevalence of lysogenic phages in the macroalgae environment may stem from: (i) the macroalgae environment is relatively stable and often dominated by certain core bacterial taxa as compared with seawater. This may allow the phages to piggyback certain bacterial taxa as they have access to these bacterial taxa over longer periods [74]. Uncontrolled lysis can lead to the complete elimination of susceptible hosts. (ii) The faster growth rate of certain bacteria like *Rhodobacteraceae*, *Maricaulaceae*, and *Sphingomonadaceae* (~5 or < 5 h of minimum doubling time, Fig. 1E) may favor lysogeny than lytic lifestyle [73]. Lysogeny benefits the macroalgae symbiotic bacteria through the exclusion of superinfection [75], protection against phagocytosis by eukaryotes [76], biofilm formation and maintenance [77], and provision of competitiveness by AMG expressions [16], likely contributing to their colonization and dominance in macroalgae. Moreover, we identified many prophage-encoded AMGs for folate biosynthesis (Fig. S1), and folate was essential for vitamin B_12_ synthesis of macroalgae [8], ensuring the growth of macroalgae. Previously such benefits for algae were solely attributed to symbiotic bacteria [8], neglecting the potential role of prophages in vitamin provision for macroalgae growth. Altogether, viruses inhabiting macroalgae likely maintain a fine-tuned equilibrium of macroalgae microbiome via lytic and lysogenic infections, suppressing excessive bacterial growth while conferring competitive advantages and potentially providing benefits for macroalgae growth by AMG expressions.

Our mesocosm experiment demonstrated that kelp cultivation had a profound impact on surrounding virioplankton communities, like increasing viral abundance, shifting viral community structures, and enriching both *Phycodnaviridae* viruses and bacteriophages. *Phycodnaviridae* viruses were primarily enriched in the cell-enriched fractions of seawater (Fig. 2G), owing to their large particle sizes (typically >0.22 μm) [78]. Similar enrichment was also observed in a diatom-dominated phytoplankton bloom [26]. Reasonably, some of these enriched *Phycodnaviridae* viruses might infect kelp which generally harbor latent viruses integrated into vegetative cells [21, 70]. However, environmental changes during the mesocosm experiments might have promoted the release of free virus particles via lysis of kelp reproductive cells (gametes and zoospores) [79] into the seawater. Additionally, some enriched *Phycodnaviridae* viruses might also infect kelp epiphytic or phycosphere algae, such as Chlorophyta (*Micromonas*, *Chlamydomonas*) or non-kelp Ochrophyta (*Ectocarpus*) [72, 80]. We acknowledge that the *Phycodnaviridae* host prediction remains challenging given the limited knowledge of these viruses [81]. Additionally, the potential eukaryotic viruses identified from seawater, as well as those originating from kelp and sediment in this study, are likely to represent free viruses rather than integrated within host genomes. This inference was supported by their close phylogenomic clustering with publicly available giant virus genomes previously discovered in planktonic communities of both marine and freshwater environments (Fig. S11). Beyond DNA viruses, previous studies have reported numerous RNA viruses infecting kelp [82] or other macroalgae-associated fungi and microalgae [22, 83]. Thus, macroalgae may also influence the dynamics of the RNA virus communities, warranting further investigation.

Although kelp cultivation significantly altered surrounding environmental conditions like increased DOC, POC, and DO, through photosynthesis, erosion, fragmentation, and grazing by herbivory [84–86], these changes predominantly influenced prokaryotic communities, rather than viral communities (Table S10). Therefore, we speculate that the changes in virioplankton communities likely stemmed from changes in prokaryotic hosts, critical for viral replication. For example, concurrent enrichment of certain bacteria and their phages were observed in this study (e.g. *Flavobacteriaceae*, *Saprospiraceae*, and *Alteromonadaceae* bacteria and their phages) and during an *Ulva prolifera* green tide (e.g. *Synechococcus* and *Roseobacter* bacteria and their phages) [18]. The proliferation of these host bacteria that thrive on algal organic matter may fuel a replication surge in their lytic phages. Additionally, lysogenic viruses also increased with kelp cultivation potentially due to a change in their infection strategy from lysogenic to lytic (e.g. *Polaribacter* phages). Similar phenomena were also observed during and after phytoplankton (diatoms and green algae) blooms [24]. Thus, the viral lifestyle transitions between lysogenic and lytic infections may be indirectly influenced by the macroalgae. Multiple factors, including nutrients, salinity, aeration, ultraviolet radiation, temperature, and host density, can affect the viral lysogenic–lytic lifestyle transition [87]. We propose that the macroalgae cultivation-driven changes in the environmental factors, especially nutrients and oxygen enrichment, likely increased prokaryotic host density and activity, triggering the lysogenic-lytic lifestyle transition of virioplanktons. This stimulates phage activities and the production of numerous progeny phage particles, typically <0.22 μm [17], which subsequently enter the virus-enriched fractions and increase the viral community diversity therein (Fig. 2C). During this phage replication and reproduction process, they also control the growth of dominant bacterial populations, maintaining prokaryotic community diversity in the macroalgae surrounding seawater.

Beyond the environmental and prokaryotic composition changes, the direct exchanges of viruses and virus-host pairs between kelp and surrounding seawater can also affect the virioplankton communities. Evidence showed that the shared viruses and virus-host pairs among the kelp and surrounding seawater environments were primarily kelp-associated bacteria (i.e. *Flavobacteriaceae* and *Rhodobacteraceae*) and their phages (Fig. 3D and E). The majority of these shared viruses with predicted lifestyles were mainly lysogenic and were identified from seawater cell-enriched fractions, suggesting that the viral migration between kelp and seawater relies on their prokaryotic hosts through lysogeny. The wide distribution of lysogens among the kelp-inhabiting *Flavobacteriaceae* and *Rhodobacteraceae* bacteria (Fig. 1E) suggests that passive transfer of viruses (especially the prophages in virocells) from kelp surface to the surrounding seawater may be substantial. However, we cannot confirm the direction of virus migration (from kelp to seawater or vice versa) due to the adhesion ability of kelp-associated bacteria to various surfaces [5, 11, 24].

Even though kelp farming primarily occurs in seawater, it can also affect the benthic viral communities in underlying sediments, especially the surface sediments, shaping an endemic viral community (Fig. 4A and B). The deposited kelp detritus may likely affect the benthic prokaryotic and eukaryotic communities, cascading to associated viruses (Supplementary Text). Additionally, benthic viral communities were also affected by the virioplanktons in the upper ocean, as reflected by the shared viruses between seawater and sediment samples (Fig. S5), suggesting the vertical transportation of viruses from seawater to sediments via the “biological pump”. Similar phenomena have been reported between seawater and sediments [59, 88], as well as between surface and deep seawater [66]. Moreover, we discovered that viruses dissociated from kelp might be directly deposited into seafloor sediments and even preserved for several decades in deep layers, based on the shared viral species and clusters (Fig. S5). We speculate that some giant viruses, like *Phycodnaviridae* viruses infecting kelp, may sink intact due to their large size or host incorporation [21, 70]. Additionally, some bacteriophages or those integrated into host cells could also descend with kelp debris or dying kelp tissues. Furthermore, we speculate that these buried viruses from kelp might not only be trapped or dormant in sediments but also undergo replication and adaptation to benthic environments. Previous research has shown that some viruses deposited in sediments from the upper ocean can retain their ability to infect susceptible hosts in the sediments [89]. Similarly, we identified a putative kelp epiphytic virus predicted to infect benthic *Woeseiaceae* bacteria, which were abundant in surface sediments of kelp farming areas (Fig. 4G) and widely distributed in global sediment environments [64]. Although this epiphytic virus was not detected in sediment metagenomes, we can not conclude the possibility that this virus can be transported into sediments along with kelp tissues and affect the dominant benthic bacteria in sediments. Consequently, kelp farming sculpts unique benthic viral communities beneath kelp farming areas that likely feed back on benthic prokaryotic communities. We must acknowledge that, in natural environments, a more diverse array of kelp-associated prokaryotes and viruses inhabiting kelp might be transported into the surrounding seawater or sediments than those studied here. This is because our study focused solely on viruses and prokaryotes from kelp blade tissues, whereas the microbial community compositions of kelp holobionts vary across various factors, including thallus regions (e.g. holdfast, stipe, meristem, blade) [10], tissue ages, and geographic locations of kelps [11].

Auxiliary metabolic genes carried by viruses can redirect host metabolism to promote replication, indirectly affecting the local biogeochemical cycling [16]. Even though viral AMGs related to carbohydrate metabolism (CAZymes) are common in organic matter-enriched environments [90, 91], their role in macroalgae systems remains underexplored. Our study reveals widespread potentially functionally active viral laminarinase AMGs involved in carbohydrate metabolism across kelp holobionts, kelp-farming seawater, and sediments of kelp-farming areas (Table S12). Laminarinases (EC 3.2.1.6, GH16) can degrade laminarin, a major kelp carbon storage and defense polysaccharide [92]. Although viral laminarinases have been recorded previously, with 186 laminarinase-like viral AMG protein sequences in the NCBI database (September 2022) (Fig. S9), the viral laminarinases identified in this study represented a new cluster of viral laminarinases encoded by macroalgae-associated phages (Fig. S9). As laminarin comprises 7–40% of kelp biomass [92], these AMGs likely persist in macroalgal viromes to assist host bacteria in utilizing the abundant organic carbon and promoting viral replication. More critically, by targeting laminarin, a key molecule in marine carbon cycling, with high annual production (12 ± 8 gigatons per year) and substantial contribution to carbon export from surface seawater [93], such virus-mediated polysaccharide hydrolysis (particularly of laminarin) may re-route substantial organic matter away from higher trophic levels. Viral metabolic manipulation of host bacteria may thus profoundly, yet cryptically, influence oceanic carbon export and energy flows.

In conclusion, our study unveiled a plethora of macroalgae-inhabiting viruses that contribute to the ecological equilibrium of macroalgal holobionts through intricate interactions. By shedding light on this cryptic role of viruses in mediating interactions between macroalgae and microorganisms, as well as revealing the shaping effects of kelp on local viral dynamics, this study lays the groundwork for future investigations into the endemic virosphere of the global macroalgae ecosystem.

## Author contributions

Y.Z. and N.J. conceived and designed the research. J.Z., Z.Z., and Z.W. conducted the study, collected the samples, and performed the metagenomic sequencing. J.Z. and S.N. analyzed the data. J.Z., S.N., and Y.Z. wrote the manuscript. All the authors have read and approved the final manuscript.

## Conflicts of interest

The authors declare no competing interests.

## Funding

This work was supported by the projects of the Natural Science Foundation of China (No. U1906216, 42188102, 42206124, and 42350410437), the Postdoctoral Fellowship Program of CPSF (No. GZC20232809), the Shandong Province Postdoctoral Fund Project (No. SDBX2022030), the Ministry of Science and Technology Foreign Youth Expert Talent Project (No. QN2022025001L), the Qingdao Postdoctoral Fund Project (No. QDBSH20240102185), and the Ocean Negative Carbon Emissions (ONCE) Program.

## Data availability

All raw sequence data generated in this study have been deposited in the NCBI’s Sequence Read Archive (SRA) database. The raw reads of 16S rRNA gene amplicon sequencing of kelp and seawater samples, kelp metagenomes, and seawater (cell-enriched and virus-enriched fractions) metagenomes can be accessed under the BioProject PRJNA1046661. The raw reads of 16S rRNA gene amplicon sequencing and metagenomes of sediment samples can be accessed under the BioProject PRJNA861850. The nucleotide sequences of all prokaryotic ASVs, pMAGs, vOTUs, and vMAGs have been deposited in figshare (https://doi.org/10.6084/m9.figshare.c.6955776.v2).

## Code availability

All scripts, network, and phylogenetic tree files used to analyze data and generate figures in this study are available in the following GitHub repository: https://github.com/Jiulong-Zhao/Kelp-associated-virosphere.

## Supplementary Material

Supplementary_Materials_clean_wrae083

Supplementary_Tables1-12_wrae08

Supplementary_Tables13-18_wrae08

## References

[1] van der Loos LM , Eriksson BK, Falcao SJ. The macroalgal holobiont in a changing sea. Trends Microbio*l* 2019;27:635–50. 10.1016/j.tim.2019.03.002 31056303

[2] Li H , Zhang Z, Xiong T et al. Carbon sequestration in the form of recalcitrant dissolved organic carbon in a seaweed (kelp) farming environment. Environ Sci Techno*l* 2022;56:9112–22. 10.1021/acs.est.2c01535 35686906

[3] Krause-Jensen D , Duarte CM. Substantial role of macroalgae in marine carbon sequestration. Nat Geosc*i* 2016;9:737–42. 10.1038/ngeo2790

[4] Suttle CA . Viruses in the sea. Natur*e* 2005;437:356–61. 10.1038/nature04160 16163346

[5] Cai L , Gao X, Saha M et al. How do epiphytic and surrounding seawater bacterial communities shift with the development of the *Saccharina japonica* farmed in the northern China? Front Mar Sc*i* 2023;10:1117926. 10.3389/fmars.2023.1117926

[6] Wang G , Chang L, Zhang R et al. Can targeted defense elicitation improve seaweed aquaculture? J Appl Phyco*l* 2019;31:1845–54. 10.1007/s10811-018-1709-6

[7] Zhang X , Chen Y, Saha M et al. *Pseudoalteromonas piscicida* X-8 causes bleaching disease in farmed Saccharina japonica. Aquacultur*e* 2022;546:737354. 10.1016/j.aquaculture.2021.737354 PMC9855529 36671739

[8] Croft MT , Lawrence AD, Raux-Deery E et al. Algae acquire vitamin B12 through a symbiotic relationship with bacteria. Natur*e* 2005;438:90–3. 10.1038/nature04056 16267554

[9] Weigel Brooke L , Miranda Khashiff K, Fogarty Emily C et al. Functional insights into the kelp microbiome from metagenome-assembled genomes. mSystem*s* 2022;7:e01422–1. 10.1128/msystems.01422-21 35642511 PMC9238374

[10] Ihua MW , FitzGerald JA, Guiheneuf F et al. Diversity of bacteria populations associated with different thallus regions of the brown alga *Laminaria digitata*. PLoS On*e* 2020;15:e0242675. 10.1371/journal.pone.0242675 33237941 PMC7688147

[11] Weigel BL , Pfister CA. Successional dynamics and seascape-level patterns of microbial communities on the canopy-forming kelps *Nereocystis luetkeana* and *Macrocystis pyrifera*. Front Microbio*l* 2019;10:346. 10.3389/fmicb.2019.00346 30863387 PMC6399156

[12] Minich JJ , Morris MM, Brown M et al. Elevated temperature drives kelp microbiome dysbiosis, while elevated carbon dioxide induces water microbiome disruption. PLoS On*e* 2018;13: e0192772. 10.1371/journal.pone.0192772 29474389 PMC5825054

[13] Zhang Y , Nair S, Zhang Z et al. Adverse environmental perturbations may threaten kelp farming sustainability by exacerbating Enterobacterales diseases. Environ Sci Techno*l* 2024;58:5796–810. 10.1021/acs.est.3c09921 38507562

[14] Suttle CA . Marine viruses - major players in the global ecosystem. Nat Rev Microbio*l* 2007;5:801–12. 10.1038/nrmicro1750 17853907

[15] Middelboe M , Brussaard CPD. Marine viruses: key players in marine ecosystems. Viruse*s* 2017;9:302. 10.3390/v9100302 29057790 PMC5691653

[16] Hurwitz BL , U'Ren JM. Viral metabolic reprogramming in marine ecosystems. Curr Opin Microbio*l* 2016;31:161–8. 10.1016/j.mib.2016.04.002 27088500

[17] Breitbart M , Bonnain C, Malki K et al. Phage puppet masters of the marine microbial realm. Nat Microbio*l* 2018;3:754–66. 10.1038/s41564-018-0166-y 29867096

[18] Han M , Sun J, Yang Q et al. Spatiotemporal dynamics of coastal viral community structure and potential biogeochemical roles affected by an *Ulva prolifera* green tide. mSystem*s* 2023;0:e01211–22. 10.1128/msystems.01211-22 PMC10134843 36815859

[19] Schroeder DC , Mckeown DA. Viruses of seaweeds. In: Hurst CJ (ed.), Studies in Viral Ecology, 2nd edn. Chichester: Wiley, 2021, 121–138.

[20] Roux S , Adriaenssens EM, Dutilh BE et al. Minimum information about an uncultivated virus genome (MIUViG). Nat Biotechno*l* 2019;37:29–37. 10.1038/nbt.4306 30556814 PMC6871006

[21] McKeown DA , Schroeder JL, Stevens K et al. Phaeoviral infections are present in macrocystis, Ecklonia and Undaria (Laminariales) and are influenced by wave exposure in Ectocarpales. Viruse*s* 2018;10:410. 10.3390/v10080410 30081590 PMC6116031

[22] Lachnit T , Thomas T, Steinberg P. Expanding our understanding of the seaweed holobiont: RNA viruses of the red alga *Delisea pulchra*. Front Microbio*l* 2016;6:1489. 10.3389/fmicb.2015.01489 26779145 PMC4705237

[23] Beattie DT , Lachnit T, Dinsdale EA et al. Novel ssDNA viruses detected in the virome of bleached, habitat-forming kelp *Ecklonia radiata*. Front Mar Sc*i* 2018;4:441. 10.3389/fmars.2017.00441

[24] Bartlau N , Wichels A, Krohne G et al. Highly diverse flavobacterial phages isolated from North Sea spring blooms. ISME J*.* 2022;16:555–68. 10.1038/s41396-021-01097-4 34475519 PMC8776804

[25] Zhang Z , Zhao H, Mou S et al. Phage infection benefits marine diatom *Phaeodactylum tricornutum* by regulating the associated bacterial community. Microb Eco*l* 2022;86:144–53. 10.1007/s00248-022-02045-1 35622094

[26] Alarcón-Schumacher T , Guajardo-Leiva S, Antón J et al. Elucidating viral communities during a phytoplankton bloom on the West Antarctic peninsula. Front Microbio*l* 2019;10:1014. 10.3389/fmicb.2019.01014 31139164 PMC6527751

[27] Martínez JM , Schroeder DC, Wilson WH. Dynamics and genotypic composition of *Emiliania huxleyi* and their co-occurring viruses during a coccolithophore bloom in the North Sea. FEMS Microbiol Eco*l* 2012;81:315–23. 10.1111/j.1574-6941.2012.01349.x 22404582

[28] John SG , Mendez CB, Deng L et al. A simple and efficient method for concentration of ocean viruses by chemical flocculation. Env Microbiol Re*p* 2011;3:195–202. 10.1111/j.1758-2229.2010.00208.x 21572525 PMC3087117

[29] Duhaime MB , Sullivan MB. Ocean viruses: rigorously evaluating the metagenomic sample-to-sequence pipeline. Virolog*y* 2012;434:181–6. 10.1016/j.virol.2012.09.036 23084423

[30] Xu H , Luo X, Qian J et al. FastUniq: a fast de novo duplicates removal tool for paired short reads. PLoS On*e* 2012;7:e52249. 10.1371/journal.pone.0052249 23284954 PMC3527383

[31] Nurk S , Meleshko D, Korobeynikov A et al. metaSPAdes: a new versatile metagenomic assembler. Genome Re*s* 2017;27:824–34. 10.1101/gr.213959.116 28298430 PMC5411777

[32] Li D , Luo R, Liu CM et al. MEGAHIT v1.0: a fast and scalable metagenome assembler driven by advanced methodologies and community practices. Method*s* 2016;102:3–11. 10.1016/j.ymeth.2016.02.020 27012178

[33] Langmead B , Salzberg SL. Fast gapped-read alignment with bowtie 2. Nat Method*s* 2012;9:357–9. 10.1038/nmeth.1923 22388286 PMC3322381

[34] Guo J , Bolduc B, Zayed AA et al. VirSorter2: a multi-classifier, expert-guided approach to detect diverse DNA and RNA viruses. Microbiom*e* 2021;9:37. 10.1186/s40168-020-00990-y 33522966 PMC7852108

[35] Ren J , Ahlgren NA, Lu YY et al. VirFinder: a novel k-mer based tool for identifying viral sequences from assembled metagenomic data. Microbiom*e* 2017;5:69. 10.1186/s40168-017-0283-5 28683828 PMC5501583

[36] Ren J , Song K, Deng C et al. Identifying viruses from metagenomic data using deep learning. Quant Bio*l* 2020;8:64–77. 10.1007/s40484-019-0187-4 34084563 PMC8172088

[37] von Meijenfeldt FAB , Arkhipova K, Cambuy DD et al. Robust taxonomic classification of uncharted microbial sequences and bins with CAT and BAT. Genome Bio*l* 2019;20:217. 10.1186/s13059-019-1817-x 31640809 PMC6805573

[38] Paez-Espino D , Pavlopoulos GA, Ivanova NN et al. Nontargeted virus sequence discovery pipeline and virus clustering for metagenomic data. Nat Proto*c* 2017;12:1673–82. 10.1038/nprot.2017.063 28749930

[39] Fu L , Niu B, Zhu Z et al. CD-HIT: accelerated for clustering the next-generation sequencing data. Bioinformatic*s* 2012;28:3150–2. 10.1093/bioinformatics/bts565 23060610 PMC3516142

[40] Nayfach S , Camargo AP, Schulz F et al. CheckV assesses the quality and completeness of metagenome-assembled viral genomes. Nat Biotechno*l* 2021;39:578–85. 10.1038/s41587-020-00774-7 33349699 PMC8116208

[41] Johansen J , Plichta DR, Nissen JN et al. Genome binning of viral entities from bulk metagenomics data. Nat Commu*n* 2022;13:965. 10.1038/s41467-022-28581-5 35181661 PMC8857322

[42] Li Z , Pan D, Wei G et al. Deep sea sediments associated with cold seeps are a subsurface reservoir of viral diversity. ISME *J* 2021;15:2366–78. 10.1038/s41396-021-00932-y 33649554 PMC8319345

[43] Weinheimer AR , Aylward FO. Infection strategy and biogeography distinguish cosmopolitan groups of marine jumbo bacteriophages. ISME J*.* 2022;16:1657–67. 10.1038/s41396-022-01214-x 35260829 PMC9123017

[44] Zhou YL , Mara P, Cui GJ et al. Microbiomes in the challenger deep slope and bottom-axis sediments. Nat Commu*n* 2022;13:1515. 10.1038/s41467-022-29144-4 35314706 PMC8938466

[45] Roux S , Emerson JB, Eloe-Fadrosh EA et al. Benchmarking viromics: an in silico evaluation of metagenome-enabled estimates of viral community composition and diversity. Peer*J* 2017;5:e3817. 10.7717/peerj.3817 28948103 PMC5610896

[46] Lu D-C , Wang F-Q, Amann RI et al. Epiphytic common core bacteria in the microbiomes of co-located green (Ulva), brown (Saccharina) and red (Grateloupia, Gelidium) macroalgae. Microbiome*.* 2023;11:126. 10.1186/s40168-023-01559-1 37264413 PMC10233909

[47] Love MI , Huber W, Anders S. Moderated estimation of fold change and dispersion for RNA-seq data with DESeq2. Genome Bio*l* 2014;15:1–21. 10.1186/s13059-014-0550-8 PMC4302049 25516281

[48] Liao H , Li H, Duan CS et al. Response of soil viral communities to land use changes. Nat Commu*n* 2022;13:6027. 10.1038/s41467-022-33771-2 36224209 PMC9556555

[49] Camargo AP , Nayfach S, Chen IMA et al. IMG/VR v4: an expanded database of uncultivated virus genomes within a framework of extensive functional, taxonomic, and ecological metadata. Nucleic Acids Re*s* 2022;51:D733–43. 10.1093/nar/gkac1037 PMC9825611 36399502

[50] Pratama AA , Bolduc B, Zayed AA et al. Expanding standards in viromics: in silico evaluation of dsDNA viral genome identification, classification, and auxiliary metabolic gene curation. Peer*J* 2021;9:e11447. 10.7717/peerj.11447 34178438 PMC8210812

[51] Bastian M , Heymann S, Jacomy M. Gephi: an open source software for exploring and manipulating networks. Proceedings of the International AAAI Conference on Web and Social Media 2009;3:361–62. 10.1609/icwsm.v3i1.13937.

[52] Jian H , Yi Y, Wang J et al. Diversity and distribution of viruses inhabiting the deepest ocean on earth. ISME *J* 2021;15:3094–110. 10.1038/s41396-021-00994-y 33972725 PMC8443753

[53] Rho M , Wu YW, Tang H et al. Diverse CRISPRs evolving in human microbiomes. PLoS Gene*t* 2012;8:e1002441. 10.1371/journal.pgen.1002441 22719260 PMC3374615

[54] Chan PP , Lowe TM. tRNAscan-SE: searching for tRNA genes in genomic sequences. Methods Mol Bio*l* 2019;1962:1–14. 31020551 10.1007/978-1-4939-9173-0_1 PMC6768409

[55] Coclet C , Roux S. Global overview and major challenges of host prediction methods for uncultivated phages. Curr Opin Viro*l* 2021;49:117–26. 10.1016/j.coviro.2021.05.003 34126465

[56] Roux S , Camargo AP, Coutinho FH et al. iPHoP: an integrated machine learning framework to maximize host prediction for metagenome-derived viruses of archaea and bacteria. PLoS Bio*l* 2023;21:e3002083. 10.1371/journal.pbio.3002083 37083735 PMC10155999

[57] Yoshikawa G , Blanc-Mathieu R, Song C et al. Medusavirus, a novel large DNA virus discovered from hot spring water. J Viro*l* 2019;93:e02130–18. 10.1128/JVI.02130-18 PMC6450098 30728258

[58] Shaffer M , Borton MA, McGivern BB et al. DRAM for distilling microbial metabolism to automate the curation of microbiome function. Nucleic Acids Re*s* 2020;48:8883–900. 10.1093/nar/gkaa621 32766782 PMC7498326

[59] Zhao J , Jing H, Wang Z et al. Novel viral communities potentially assisting in carbon, nitrogen, and sulfur metabolism in the upper slope sediments of Mariana trench. mSystems*.* 2022;7:e01358–21. 35089086 10.1128/msystems.01358-21 PMC8725595

[60] Sullivan MJ , Petty NK, Beatson SA. Easyfig: a genome comparison visualizer. Bioinformatic*s* 2011;27:1009–10. 10.1093/bioinformatics/btr039 21278367 PMC3065679

[61] Camargo AP , Roux S, Schulz F et al. Identification of mobile genetic elements with geNomad. Nat Biotechno*l* 2023. 10.1038/s41587-023-01953-y PMC11324519 37735266

[62] Kieft K , Zhou Z, Anantharaman K. VIBRANT: automated recovery, annotation and curation of microbial viruses, and evaluation of viral community function from genomic sequences. Microbiome*.* 2020;8:90. 10.1186/s40168-020-00867-0 32522236 PMC7288430

[63] Parras-Molto M , Rodriguez-Galet A, Suarez-Rodriguez P et al. Evaluation of bias induced by viral enrichment and random amplification protocols in metagenomic surveys of saliva DNA viruses. Microbiome*.* 2018;6:119. 10.1186/s40168-018-0507-3 29954453 PMC6022446

[64] Hoffmann K , Bienhold C, Buttigieg PL et al. Diversity and metabolism of Woeseiales bacteria, global members of marine sediment communities. ISME J*.* 2020;14:1042–56. 10.1038/s41396-020-0588-4 31988474 PMC7082342

[65] Ye N , Zhang X, Miao M et al. Saccharina genomes provide novel insight into kelp biology. Nat Commu*n* 2015;6:6986. 10.1038/ncomms7986 25908475 PMC4421812

[66] Zhao J , Wang Z, Li C et al. Significant differences in planktonic virus communities between “cellular fraction” (0.22 ~ 3.0 μm) and “viral fraction” (< 0.22 μm) in the ocean. Microb Eco*l* 2022;86:825–42. 10.1007/s00248-022-02167-6 36585490

[67] Cheng R , Li X, Jiang L et al. Virus diversity and interactions with hosts in deep-sea hydrothermal vents. Microbiome*.* 2022;10:235. 10.1186/s40168-022-01441-6 36566239 PMC9789665

[68] Zhou Y-L , Mara P, Vik D et al. Ecogenomics reveals viral communities across the challenger deep oceanic trench. Commun Bio*l* 2022;5:1055. 10.1038/s42003-022-04027-y 36192584 PMC9529941

[69] Moniruzzaman M , Weinheimer AR, Martinez-Gutierrez CA et al. Widespread endogenization of giant viruses shapes genomes of green algae. Natur*e* 2020;588:141–5. 10.1038/s41586-020-2924-2 33208937

[70] Van Etten JL , Dunigan DD, Nagasaki K, et al. Phycodnaviruses (Phycodnaviridae). In: Bamford DH, Zuckerman M (eds). Encyclopedia of Virolog*y* (Fourth Edition). Academic Press: Oxford, 2021, pp 687-695. 10.1016/B978-0-12-809633-8.21291-0

[71] Olga Maria L , Ana Patrícia G. Biofilms: an extra coat on macroalgae. In: Nooruddin T, Dharumadurai D (ed.), Algae - Organisms for Imminent Biotechnology. Rijeka: IntechOpen, 2016, 183–210.

[72] Liu Y , Wikfors GH, Clark P et al. A deep dive into the epibiotic communities on aquacultured sugar kelp *Saccharina latissima* in southern New England. Algal Re*s* 2022;63:102654. 10.1016/j.algal.2022.102654

[73] Yi Y , Liu S, Hao Y et al. A systematic analysis of marine lysogens and proviruses. Nat Commu*n* 2023;14:6013. 10.1038/s41467-023-41699-4 37758717 PMC10533544

[74] Nair S , Li C, Mou S et al. A novel phage indirectly regulates diatom growth by infecting a diatom-associated biofilm-forming bacterium. Appl Environ Microbio*l* 2022;88:e02138–21. 10.1128/aem.02138-21 35020448 PMC8904054

[75] Bondy-Denomy J , Qian J, Westra ER et al. Prophages mediate defense against phage infection through diverse mechanisms. ISME J*.* 2016;10:2854–66. 10.1038/ismej.2016.79 27258950 PMC5148200

[76] Brüssow H . Bacteria between protists and phages: from antipredation strategies to the evolution of pathogenicity. Mol Microbio*l* 2007;65:583–9. 10.1111/j.1365-2958.2007.05826.x 17608793

[77] Obeng N , Pratama AA, Elsas JDv. The significance of mutualistic phages for bacterial ecology and evolution. Trends Microbio*l* 2016;24:440–9. 10.1016/j.tim.2015.12.009 26826796

[78] Needham DM , Yoshizawa S, Hosaka T et al. A distinct lineage of giant viruses brings a rhodopsin photosystem to unicellular marine predators. Proc Natl Acad Sci US*A* 2019;116:20574–83. 10.1073/pnas.1907517116 31548428 PMC6789865

[79] Miiller DG . Marine virioplankton produced by infected *Ectocarpus siliculosus* (Phaeophyceae). Mar Ecol Prog Se*r* 1991;76:101–2. 10.3354/meps076101

[80] Sanjaya EH , Hidayati EDH, Prabaningtyas S et al. Potential of microalgae isolate kelp I (Brawijaya museum pond) and isolate kelp IV (Selorejo reservoir) as biodiesel feedstock. AIP Conference Proceeding*s* 2021;2353:030074.

[81] Cheng S , Wong GK, Melkonian M. Giant DNA viruses make big strides in eukaryote evolution. Cell Host Microb*e* 2021;29:152–4. 10.1016/j.chom.2021.01.008 33571441

[82] Easton LM , Lewis GD, Pearson MN. Virus-like particles associated with dieback symptoms in the brown alga Ecklonia radiata. Dis Aquat Or*g* 1997;30:217–22. 10.3354/dao030217

[83] Lang AS , Culley AI, Suttle CA. Genome sequence and characterization of a virus (HaRNAV) related to picorna-like viruses that infects the marine toxic bloom-forming alga Heterosigma akashiwo. Virolog*y* 2004;320:206–17. 10.1016/j.virol.2003.10.015 15016544

[84] Weigel BL , Pfister CA. The dynamics and stoichiometry of dissolved organic carbon release by kelp. Ecolog*y* 2021;102:e03221. 10.1002/ecy.3221 33048348

[85] Feng X , Li H, Zhang Z et al. Microbial-mediated contribution of kelp detritus to different forms of oceanic carbon sequestration. Ecol Indi*c* 2022;142:109186. 10.1016/j.ecolind.2022.109186

[86] Li H , Feng X, Xiong T et al. Particulate organic carbon released during macroalgal growth has significant carbon sequestration potential in the ocean. Environ Sci Techno*l* 2023;57:19723–31. 10.1021/acs.est.3c04959 37963337

[87] Zhang M , Zhang T, Yu M et al. The life cycle transitions of temperate phages: regulating factors and potential ecological implications. Viruses*.* 2022;14:1904. 10.3390/v14091904 36146712 PMC9502458

[88] Wang L , Zhao J, Wang Z et al. phoH-carrying virus communities responded to multiple factors and their correlation network with prokaryotes in sediments along Bohai Sea, Yellow Sea, and East China Sea in China. Sci Total Enviro*n* 2022;812:152477. 10.1016/j.scitotenv.2021.152477 34952046

[89] Vincent F , Vardi A. Viral infection in the ocean—a journey across scales. PLoS Bio*l* 2023;21:e3001966. 10.1371/journal.pbio.3001966 36701270 PMC9879395

[90] Jin M , Guo X, Zhang R et al. Diversities and potential biogeochemical impacts of mangrove soil viruses. Microbiome*.* 2019;7:58. 10.1186/s40168-019-0675-9 30975205 PMC6460857

[91] Bi L , Yu DT, Du S et al. Diversity and potential biogeochemical impacts of viruses in bulk and rhizosphere soils. Environ Microbio*l* 2021;23:588–99. 10.1111/1462-2920.15010 32249528

[92] Sterner M , Gröndahl F. Extraction of laminarin from *Saccharina latissima* seaweed using cross-flow filtration. J Appl Phyco*l* 2021;33:1825–44. 10.1007/s10811-021-02398-z

[93] Becker S , Tebben J, Coffinet S et al. Laminarin is a major molecule in the marine carbon cycle. Proc Natl Acad Sci US*A* 2020;117:6599–607. 10.1073/pnas.1917001117 32170018 PMC7104365

