# Supplementary material for: Macroalgal virosphere assists with host–microbiome equilibrium regulation and affects prokaryotes in surrounding marine environments
Source: ISME J. 2024 May 6;18(1):wrae083. doi: 10.1093/ismejo/wrae083 (PMC11126160; doi:10.1093/ismejo/wrae083)
Supplement: Supplementary_Materials_clean_wrae083 [file supplementary_materials_clean_wrae083.docx]

**Supplementary Materials for**

**Macroalgal virosphere assists with host-microbiome equilibrium regulation and affects prokaryotes in surrounding marine environments**

**Running Title: Exploration of macroalgal virosphere**

Jiulong Zhao^1,2,3†^, Shailesh Nair^1,2,3†^, Zenghu Zhang^1,2,3,4^, Zengmeng Wang^1,2,3^, Nianzhi Jiao^5^, Yongyu Zhang^1,2,3,4^*

^1^Qingdao Institute of Bioenergy and Bioprocess Technology, Chinese Academy of Sciences, Qingdao, China

^2^Shandong Energy Institute, Qingdao, Shandong, China

^3^Qingdao New Energy Shandong Laboratory, Qingdao, China

^4^University of Chinese Academy of Sciences, Beijing, China

^5^Institute of Marine Microbes and Ecospheres, State Key Laboratory of Marine Environmental Science, Xiamen University, Xiamen, China

^†^ Authors contributed equally to this work.

*Corresponding Authors.

E-mail: Y.Z. zhangyy@qibebt.ac.cn;

**Supplementary Materials**

**Supplementary Text**

**Profiles of viral communities and functions identified from kelp, seawater, and sediments**

From metagenomic assemblies of seawater [cell-enriched fraction (>0.2 μm) and virus-enriched fraction (< 0.2 μm)] samples, kelp (tissue and surface mucus) samples, and sediment samples, we recovered a total of 39,379 vOTUs (>5 kb) representing the most realistic virus community profiles using multiple viral identification tools and stringent criteria (Table S13).

Of these, 5,570 vOTUs were identified from kelp metagenomes (vOTUs_k_, Table S1), delineating an approximately species-level viral community. The vOTUs_k_ ranged in size from 5,001 to 94,853 bp, with 108 of them having >50% genome completeness and no contamination. 47 of these were of high quality and near-complete (>90% completeness) genomes. Viral binning of the kelp metagenomes yielded 83 metagenome-assembled genomes (vMAGs_k_), ranging from 5,127 to 346,548 bp in size (Table S2).

From seawater samples of cell-enriched fractions and virus-enriched fractions, we obtained a total of 12,518 vOTUs (vOTUs_sw_). 723 of them had > 50% completeness and no contamination, including 532 near-complete (>90% completeness) genomes (Table S1). Viral binning produced 632 vMAGs_sw_ with >90% completeness and no contamination. These comprised 63 and 569 vMAGs_sw_ from cell-enriched and virus-enriched fractions, respectively, and ranging in size from 5,007 to 685,580 bp (Table S2). According to the PCoA and ADONIS analyses, CF and VF viral community structures were significantly distinct (ADONIS: *R* = 0.454, *p* < 0.01, Fig. S5d), with only 14.4% (1,803 out of 12,518) shared vOTUs. Whereas a significant portion of vOTUs_sw_ were taxonomically unclassified (~69.8%), the assigned vOTUs predominantly belonged to bacteriophages from the Caudovirales order (18.8%, i.e., *Myoviridae*, *Siphoviridae*, *Podoviridae*, and unclassified *Caudovirales*) (Fig. 2e). For vMAGs_sw_, 64.2% of them were taxonomically classified, and those belonging to Phixviricota (*Microviridae*) account the highest proportion (Fig. 2e). Additionally, 9.6% of all vOTUs_sw_ and 2.1% of all vMAGs_sw_ potentially belonged to eukaryotic viruses originating from the Nucleocytoviricota phylum (Fig. 2e). According to the host prediction, ~14.3% (1,792 out of 12,518) of vOTUs_sw_ and ~7.1% (45 out of 632) of vMAGs_sw_ were linked to potential prokaryotic hosts, primarily belonging to Bacteroidia (linked to ~7.2% vOTUs_sw_ and ~2.8% vMAGs_sw_), Alphaproteobacteria (~2.4% and ~0.8%), and Gammaproteobacteria (~2.1% and ~1.7%). A gene-sharing network revealed 979 viral clusters (VCs), comprising 341 vOTUs(/vMAGs)_k_ and 2,759 KSW-vOTUs(/vMAGs)_sw_. 17 of these VCs contained viral genomes from both kelp and seawater environments (Fig. 4e & S5c).

Sediment viral communities comprised 21,291 vOTUs (vOTUs_sd_) dereplicated at the species level (Table S1). vOTUs_sd_ ranged in size from 5,001 to 168,588 bp, with 808 of them with completeness >50% and zero contamination, including 199 near-complete (completeness >90%) viral genomes. 369 vMAGs were assembled from the sediment metagenomes (vMAGs_sd_), ranging from 5,205 to 464,168 bp in size (Table S2).

We functionally annotated viral open reading frames (ORFs) from kelp, seawater, and sediment vOTUs using the KEGG database by KofamScan v1.3.0 (-E 0.00001) [1]. Only 5.0% (2,380 out of 47,922), 7.3% (11,807 out of 162,838), and 5.6% (17,172 out of 307,543) of ORFs predicted from vOTUs_k_, vOTUs_sw_, and vOTUs_sd_, respectively, were annotated. Among annotated viral proteins, predominant functions included “Genetic information processing”, “Replication and repair”, “Nucleotide metabolism” categories (Fig. S12), which are known to be critical for virus reproduction. Viral ORFs annotated under the “Carbohydrate metabolism” category, which might include many viral auxiliary metabolic genes (AMGs), were also abundant.

**Endophytic and epiphytic prokaryotic communities inhabiting kelp**

The most dominant bacteria inhabiting kelp were those belonging to *Flavobacteriaceae* (mainly *Algitalea*, *Winogradskyella*, and *Tenacibaculum* genera), *Rhodobacteraceae* (mainly *Yoonia-Loktanella*, *Sulfitobacter*, and *Octadecabacter* genera), and *Hyphomonadaceae* (mainly *Litorimonas* and *Fretibacter* genera) family (Table S14). These dominant bacteria were primarily epiphytic, whereas only a few bacteria were potential endophytic, like some *Enterobacteriaceae*, *Pseudomonadaceae* and *Streptomycetaceae* bacteria species (Table S15). Similar bacterial compositions in kelp were also reported in previous studies [2-4]. Meanwhile, previous studies also revealed that epiphytic bacteria composition differs across kelp species [5].

**Shaping effects of kelp cultivation on bacterioplankton communities**

Although comparable alterations in alpha diversity (Chao1 and Shannon) of prokaryotic communities were noticed in control and kelp-inoculated groups after 63 h (Fig. S4a-b), significant dissimilarities in beta diversity (PCoA with PERMANOVA, Fig. S4c) pointed to distinctive prokaryotic community structure in kelp inoculated treatments. After 63 hours, *Rhodobacteraceae*, *Saprospiraceae*, *Alteromonadaceae*, and *Flavobacteriaceae* dominated the kelp mesocosms, whereas *Rhodobacteraceae*, *Litoricolaceae*, *SAR11_Clade_I*, *Flavobacteriaceae*, *Halieaceae*, and *Microbacteriaceae* were dominant in control group (Fig. S4d & Table S16). Correlation analysis revealed that multiple environmental changes significantly drove the changes in bacterial community compositions (Fig. S4e). Differential abundance analysis revealed more ASVs were significantly altered in the kelp mesocosms after 63-h experiment (Fig. S4f-g & Table S17), particularly enrichment of ASVs belonging to the families of *Rhodobacteraceae*, *Flavobacteriaceae* (mainly *Polaribacter* genus), *Alteromonadaceae*, and *Marinomonadaceae* (mainly *Marinomonas* genus) (Fig. S4h). These groups are previously reported to be abundant as macroalgal epiphytes or within macroalgal phycosphere and are sensitive to macroalgae growth dynamics [6-11]. For example, *Polaribacter*, known to degrade algal polysaccharides like alginate, agar and carrageenan, likely proliferated in response to the secretion of the macroalgal organic matter [12, 13].

**Kelp farming contributes to the unique prokaryotic and viral communities in the seafloor surface sediments**

We identified 8,924 ASVs (Table S18) and 257 pMAGs (named pMAGs_sd_, Table S4) to profile the prokaryotic communities in sediments. The benthic prokaryotic community structures differed markedly from those in kelp or seawater, with dominance of the members of the phyla Chloroflexi (average 20.2% across 9 samples), Planctomycetota (15.2%), Crenarchaeota (12.9%) (classified as Thermoproteota phylum in GTDB database) [14], and Desulfobacterota (11.0%) (Fig. S13c). The surface sediments beneath the high-density kelp farming area (S9) displayed a high degree of endemicity in the prokaryotic community based on the clustering and PCoA analyses (Fig. S13b).

Comparing only the surface sediments, the higher deposition of kelp-released organic matter (including kelp debris, naturally detached old blades, and fragmentized normal blades) [15, 16], containing specific organic carbon compounds, may have reshaped the benthic prokaryotic communities. This is evidenced through the enrichment of Gammaproteobacteria (mainly *Woeseiaceae* family) and lower community richness and evenness (Chao1 and Shannon indices) compared to other sediment samples (Fig. S13a, c). Such shaping effects have been previously observed in sediments from kelp and *Gracilaria* cultivation areas [10, 17]. The higher enrichment of members of the *Woeseiaceae* family (Fig. S14) further corroborates their capability for the degradation and utilization of algal polymers (including polysaccharides and detrital proteins) [18-21]. Although other eukaryotes (including benthic protists and flora and fauna) were not studied here, they were also reported to be affected by macroalgal cultivation [22]. Therefore, no matter whether prokaryotes or eukaryotes, their associated viruses in sediments, would be expected to respond to the changes in benthic biocoenosis [22], especially viruses in surface sediments beneath the kelp farming areas displaying significantly endemic prokaryotic communities (Fig. 4a-b).

**Supplementary Methods**

**Abiotic and biotic factor measurements of the seawater in the mesocosm experiment**

To monitor the changes in abiotic and biotic factors during the mesocosm experiment, seawater samples were collected from the enclosures three times a day (at hours 0, 15, 20, 24, 39, 44, 48 and 63, Table S9). All abiotic factors of seawater were measured as previously reported [23]. Briefly, the seawater temperature, salinity, and dissolved oxygen (DO) were determined by the water quality analyzer (EXO-2, YSI Inc., USA). Total alkalinity (TAlk) was determined by an automatic potentiometric titrator (T960E, Hanon Co., China). Dissolved organic carbon concentrations (DOC) were quantified via the high-temperature catalytic oxidation method using a TOC-L analyzer (TOC-L CPH; Shimadzu, Japan) [24]. The dissolved inorganic carbon (DIC) concentration was measured using an infrared CO_2_ detector-based DIC analyzer (AS-C5; Apollo SciTech Inc., USA) [25]. Seawater pH was calculated from temperature, salinity, TAlk, and DIC using CO2SYS.XLS (version 24) as previously performed [23]. The particulate organic carbon (POC) concentration was determined using an elemental analyzer (ECS 4024 CHNS-O NC, Technologies, Italy) [26]. Nutrient levels of NO_2_^-^, NO_3_^-^, NH_4_^+^, PO_4_^3-^, and SiO_3_^2-^ were determined by an autoanalyzer (QuAAtro 39, Seal, Germany) with detection limits of 0.06, 0.02, 0.09, 0.03, and 0.05 μM, respectively. All physicochemical parameters were measured in triplicate with the mean value calculated after excluding outlier values.

The biotic factors, mainly the prokaryotic and viral abundance, were determined using the previously described methods [27, 28]. In brief, frozen samples were thawed at 37 °C and diluted in 0.02-μm filtered Tris-EDTA buffer (pH = 8, Sigma-Aldrich) and then stained with the SYBR Green Ι (final concentration of 0.5 × 10^-4^ of the Molecular Probes stock solution, Thermo Fisher). The virus-like particle and prokaryote counts were determined using a FACSAria ΙΙ flow cytometer (BD Biosciences, USA).

**DNA extraction and sequencing**

Total genomic DNA of seawater cell-enriched fractions (> 0.2 μm) was extracted using the MagPure Soil DNA LQ Kit (Magan, China) and DNeasy PowerSoil Pro Kit (QIAGEN, Germany), from kelp tissue and surface mucus samples with the Plant DNA Extraction Mini Kit B (Mabio, China) and Advanced Soil DNA Kit (mCHIP BioTech, China), from sediment samples by MagPure Soil DNA LQ Kit (Magen, China) and Dneasy PowerSoil Kit (QIAGEN, Germany) and viral particles in seawater viral fractions (< 0.2 μm) by TaKaRa MiniBEST Viral RNA/DNA Extraction Kit Ver.5.0 (TaKaRa, China). Extractions were performed following the respective manufacturer’s instructions.

For 16S rRNA gene amplicon sequencing, the V3-V4 region from triplicate samples was PCR amplified using primers 338F (5’-GTGCCAGCMGCCGCGG-3’) and 806R (5’-GGACTACHVGGGTWTCTAAT-3’) or the V4 region for sediment samples using primers 515F (5’-GTGCCAGCMGCCGCGG-3’) and 806R (5’-GGACTACHVGGGTWTCTAAT-3’). The thermocycling conditions were as follows: 94 °C for 5 min; 26 cycles of 94 °C for 30 s, 56 °C for 30 s, 72 °C for 20 s; 72 °C for 5 min. The PCR products were purified with AMPure XP beads (Beckman Coulter, USA), followed by an additional PCR amplification round. Final amplicons were quantified using the Qubit dsDNA Assay Kit (Thermo Fisher Scientific, USA). High-throughput sequencing of amplicons was performed on the Illumina NovaSeq 6000 platform (OE Biotech Co., Ltd., Shanghai, China), producing 250 bp paired-end reads.

For metagenomic analysis, sequencing libraries were prepared using NEXTflex® Rapid DNA-Seq Kit (Bioo Scientific, USA) following the manufacturer’s instructions. For viromics, whole viral genomes underwent multiple displacement amplification using the Illustra™ Ready-To-Go GenomiPhi V3 DNA Amplification Kit (GE Healthcare, USA) following the manufacturer’s protocols. Libraries were then prepared using NEB Next^®^ Ultra™ DNA Library Prep Kit for Illumina^®^ (New England Biolabs, USA). Both metagenomic and viromic sequencing were performed on the Illumina NovaSeq 6000 platform (Magigene Biotech. Co., Ltd., Guangzhou, China), producing 150 bp paired-end reads.

**Prokaryotic community analysis through 16S rRNA gene amplicon analysis**

DADA2 v1.14 was used to differentiate the 16S rRNA gene amplicon sequence variants (ASVs), remove chimeras and assign taxonomy using Silva v138.1 database with default settings [29]. The generated ASV table was then fed into the MicrobiomeAnalyst web-based package (https://microbiomeanalyst.ca) [30] for statistics and visualization using the settings previously described [31]. Due to library construction failure, one kelp surface sample (Table S14) could not be sequenced for 16S rRNA gene amplicon. Therefore, the metagenome of this sample was used to infer bacterial composition using Kraken2 v2.1.2 [32] and Bracken v2.6.2 [33] according to the published analysis protocol [34].

**Recovery of prokaryotic metagenome-assembled genomes (pMAGs)**

Prokaryotic metagenome-assembled genomes (pMAGs) were recovered for the individual metagenomic assemblies using metaBAT2 [35], MaxBin2 [36], and CONCOCT [37] binning modules of the metaWRAP pipeline [38]. The resultant prokaryotic bins from all samples were merged, and the redundant (clustered at 95% average nucleotide identity) and low-quality (completeness < 50% and contamination rate > 10%) bins were removed using dRep v2.6.2 [39]. To strongly avoid the virus-host prediction errors linked to pMAG contamination, the contigs in pMAGs generated by the inaccurate binning were removed as previously reported [40, 41]. Specifically, contigs with conflicting phylum-level taxonomy annotated by CAT v8.22 [42] were removed. Besides, contigs in pMAGs that carried viral region longer than 2/3rd of the contig length were discarded using BLASTN alignment against the viral sequences identified in this study (> 90% identity, > 500 bp alignment length, and > 66.67% alignment fraction relative to the contigs). The final pMAG taxonomy was assigned using the GTDB-Tk v1.3.0 toolkit [43].

**Recovery of** **viral metagenome-assembled genomes (vMAGs)**

Viral metagenome-assembled genomes (vMAGs) were binned from each metagenome using PHAMB [44]. Prokaryotic cotamination was removed through CheckV v0.7.0 [45] and high-quality (completeness > 90%) vMAGs based on CheckV’s AAI evaluation were retained for further analysis. Concurrently, high-quality (completeness > 90%) vOTUs (see main text for information) were merged with the above-filtered vMAGs and dereplicated at the species level [44] using dRep v2.6.2 (--S_algorithm fastANI, -sa 0.95, --ignoreGenomeQuality) [39]. Whenever required, contigs from individual vMAGs were stitched together with a linker sequence “NNNNCATTCCATTCATTAATTAATTAATGAATGAATGNNNNN”, to encourage proper gene predictions near the contig junctions [46].

**Viral taxonomic and lifestyle assignments**

Viral taxonomic assignments were performed on vOTUs and vMAGs with a sequence length of ≥ 10 kb or ≥ 90% genome completeness as described previously [47]. The remaining vOTUs and vMAGs were assigned as “Unclassified”. Two tools were used for taxonomic assignment: (1) Gene-sharing network-based vConTACT v2 [48] using default parameters and the NCBI RefSeq Virus database (v210), which was considered the “gold standard” of viral classification in this study and (2) protein family-based VPF-Class tool with default parameters [49]. For the VPF-Class tool, taxonomic assignments with a membership ratio (MR) of ≥ 0.5 and confidence score (CS) of ≥ 0.2, representing ~100% accuracy [47, 49] were retained. Sequences with an MR of < 0.5 and ≥ 0.2 and CS ≥ 0.2 were taxonomically assigned according to the shared lowest common ancestor (LCA) of the annotated taxonomies. In addition, ViralRecall v2.0 (-s 2) [50] and NCLDV-detector [51] with the thresholds of “Pred_complex_best = ‘Virus_NCLDV’ AND Ratio_TATATA_3.6 > 0.3 AND Pred_simple_NCLDV_score > 0.3 AND Pred_complex_NCLDV_score > 0.3” were used to determine the taxonomy of NCLDVs. Finally, all classification results of vOTUs and vMAGs were merged and manually curated from domain to family ranking.

The lysogenic marker proteins of all vOTUs and vMAGs were identified using hmmsearch v3.3.1 (-E 1.0e-05; http://hmmer.org/) against the HMM database of lysogenic marker proteins (transposase, integrase, excisionase, resolvase, and recombinase proteins downloaded from Pfam v35.0) [52] to identity potential lysogenic viruses. Additionally, PhaTYP online server [53] was also used to predict the lifestyle (virulent or temperate) of viruses represented by vOTUs and vMAGs in this study.

**Calibration of genome quality and taxonomic and lifestyle assignments of NCLDVs**

Given that the smallest NCLDV genome is 103 kb [54], all NCLDV vOTUs with a size below 100 kb were considered “Genome fragments”, and those larger than 100 kb were considered “Genomes” (Table S1). Among the vMAGs dataset, NCLDV vMAGs below 100 kb in size were removed from further analysis (Table S2).

The calibration of taxonomic assignments of NCLDVs was performed through a benchmarked phylogenomic analysis based on a concatenated seven gene marker set (SFII, RNAPL, PolB, TFIIB, TopoII, A32, and VLTF3 genes) specific to Nucleocytoviricota [55]. In detail, the above marker genes were identified within the NCLDVs vOTUs/vMAGs from our study, as well as in reference Giant Virus Database (GVDB) [55]. The identified sequences were concatenated and aligned using a custom script ncldv_markersearch [54], followed by trimming using trimAl (-gt 0.1). The phylogenetic tree was inferred with the FastTree v2.0 (-gamma) [56] and visualized using iTOL (v6) [57]. The taxonomy assignments of our vOTUs/vMAGs were manually curated according to their topological positioning relative to the reference virus sequences in the phylogenomic tree [55].

Additionally, as reliable methods for predicting NCLDV lifestyles are currently lacking, their lifestyles were manually annotated as “uncertain”.

**Phylogenetic constructions of head-tail phages and NCLDVs**

A viral group-specific marker, the *terL* gene for *Caudovirales* [58], was used to perform the phylogenetic analysis of head-tail phages. All terminase large subunit HMM profiles (VOG00023, VOG00115, VOG00127, VOG00128, and VOG00290) were compiled from the VOG database (release 215). Proteins of all vOTUs and vMAGs were searched against these databases using hmmsearch (-E 1e-5). Only the best hit (highest bitscore) encoded by a vOTU or a vMAG was retained. Best hit TerL protein sequences with less than 170 aa were discarded [59], resulting in 855 and 98 TerL proteins from vOTUs and vMAGs, respectively. These TerL sequences were aligned with MAFFT v7.471 (-linsi) [60] and trimmed with trimal v1.4.rev15 (-gt 0.1) [61]. IQ-tree [62] was used to construct a maximum-likelihood phylogenetic tree using the best-fit model suggested by ModelFinder [63] and ultrafast bootstrap. The tree was visualized using iTOL (v6) [57].

To assess the phylogeny of the NCLDVs identified in this study, we constructed a phylogenetic tree using concatenated alignments of five conserved NCLDV marker genes present in representative vOTUs and vMAGs: the Major Capsid Protein (MCP, GVOGm0003), Superfamily II Helicase (SFII, GVOGm0013), Virus-like Transcription Factor (VLTF3, GVOGm0890), DNA Polymerase B (PolB, GVOGm0054), and Packaging ATPase (A32, GVOGm0760) [54]. Homologous proteins of these five genes were identified in the vOTUs and vMAGs and concatenated and aligned using a custom script ncldv_markersearch [54], followed by trimming using trimAl (-gt 0.1). The phylogenetic tree was inferred with the IQ-tree, as was performed for the TerL tree.

**Functional annotation and phylogenetic analysis of viral AMGs**

In AMG-carrying viral contigs (including vOTUs and vMAGs), the Sigma-70 transcriptional promoter was recognized by SAPPHIRE (*P* < 0.005, https://sapphire.biw.kuleuven.be/index.php). Rho-independent and Rho-dependent terminators were predicted by FindTerm (energy threshold value < -12.0, http://www.softberry.com/berry.phtml?topic=findterm&group=programs&subgroup=gfindb) and RhoTermPredict (default parameters) [64], respectively. In AMGs encoded proteins, conserved residues and active sites were identified using the NCBI CD-search tool (E-value < 1e-5) [65] and PROSITE (release 2022_03 of 03-Aug-2022, default settings, https://prosite.expasy.org/). Besides, viral protein structural predictions were conducted with Phyre2 v2.0 (http://www.sbg.bio.ic.ac.uk/phyre2/html/page.cgi?id=index), and the predicted three-dimensional structures of viral proteins were visualized online.

Phylogenetic analysis of viral AMGs was performed to investigate their evolutionary origin. Amino acid sequences of high-confidence AMGs were compared with the NCBI NR database (blastp, e-value < 1e-5) to recruit closely related sequences. The top 20 hits per query were extracted, dereplicated based on the protein accession number, and merged with query sequences. The phylogenetic trees of AMGs were constructed and visualized as performed in the TerL tree described above.

**Phylogenetic constructions of publicly available viral laminarinases**

Amino acid sequences of 186 viral laminarinases deposited in the NCBI database were retrieved and merged with sequences of 17 viral laminarinases identified in this study. All these proteins were aligned and trimmed, and the maximum-likelihood phylogenetic tree was inferred with the IQ-tree as described above.

**Supplementary Figures**

**
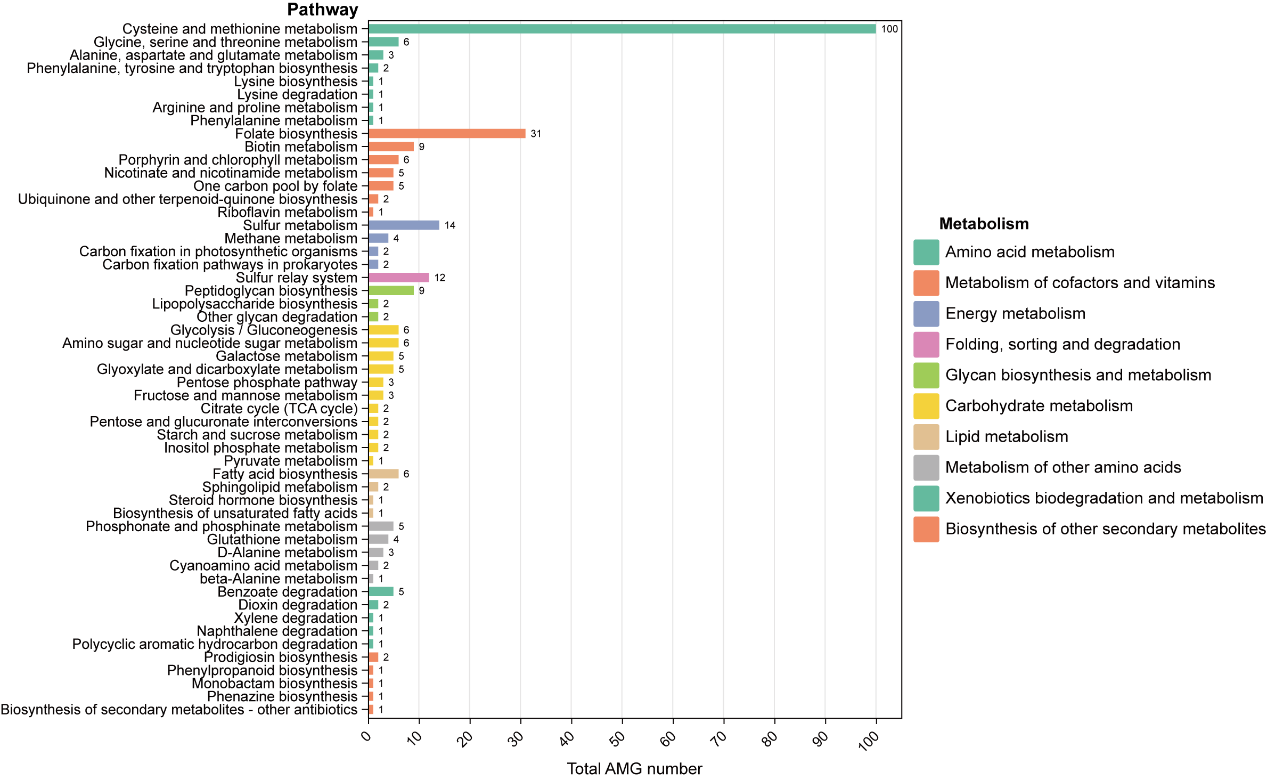
**

**Fig. S1: AMG abundance profile from prophages of macroalgae symbiont bacteria.** Barplot showing the total number of AMGs related to different KEGG pathways identified from the prophages in the macroalgae symbiotic bacterial genomes from our study and a previous study [3].


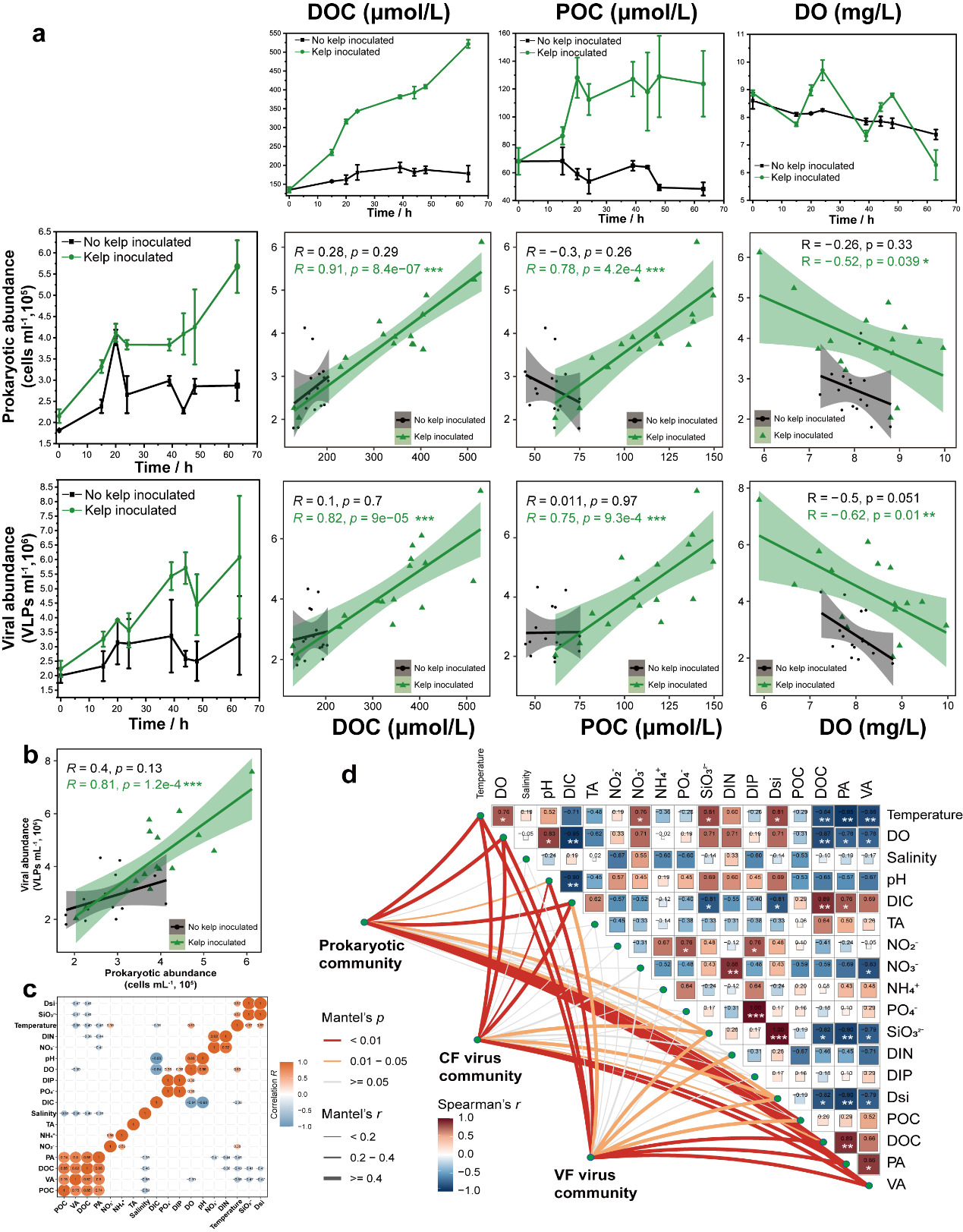


**Fig. S2: Dynamics of abiotic and biotic factors and their correlations with microbial communities during the mesocosm experiment. (a)** Changes over time in abiotic (DOC, POC, and DO concentration) and biotic (prokaryotic and viral abundance) factors and the correlations between them. The colored areas show 95% confidence intervals for the linear regression model. **(b)** The correlation between viral and prokaryotic abundances. **(c)** Correlation analysis between all abiotic and biotic factors. **(d)** Mantel test showing the correlation between abiotic and biotic factors and prokaryotic community (top node), CF viral community (middle node), and VF viral community (bottom node). The *p* values of the mantel test were adjusted using a false discovery rate (FDR). The edges connecting prokaryotic and CF and VF virus communities depict the corresponding distance correlations between communities and environmental factors inferred through the Mantel test. The width and color of these edges correspond to Mantel’s r statistic and statistical significance (*P*-value), respectively.


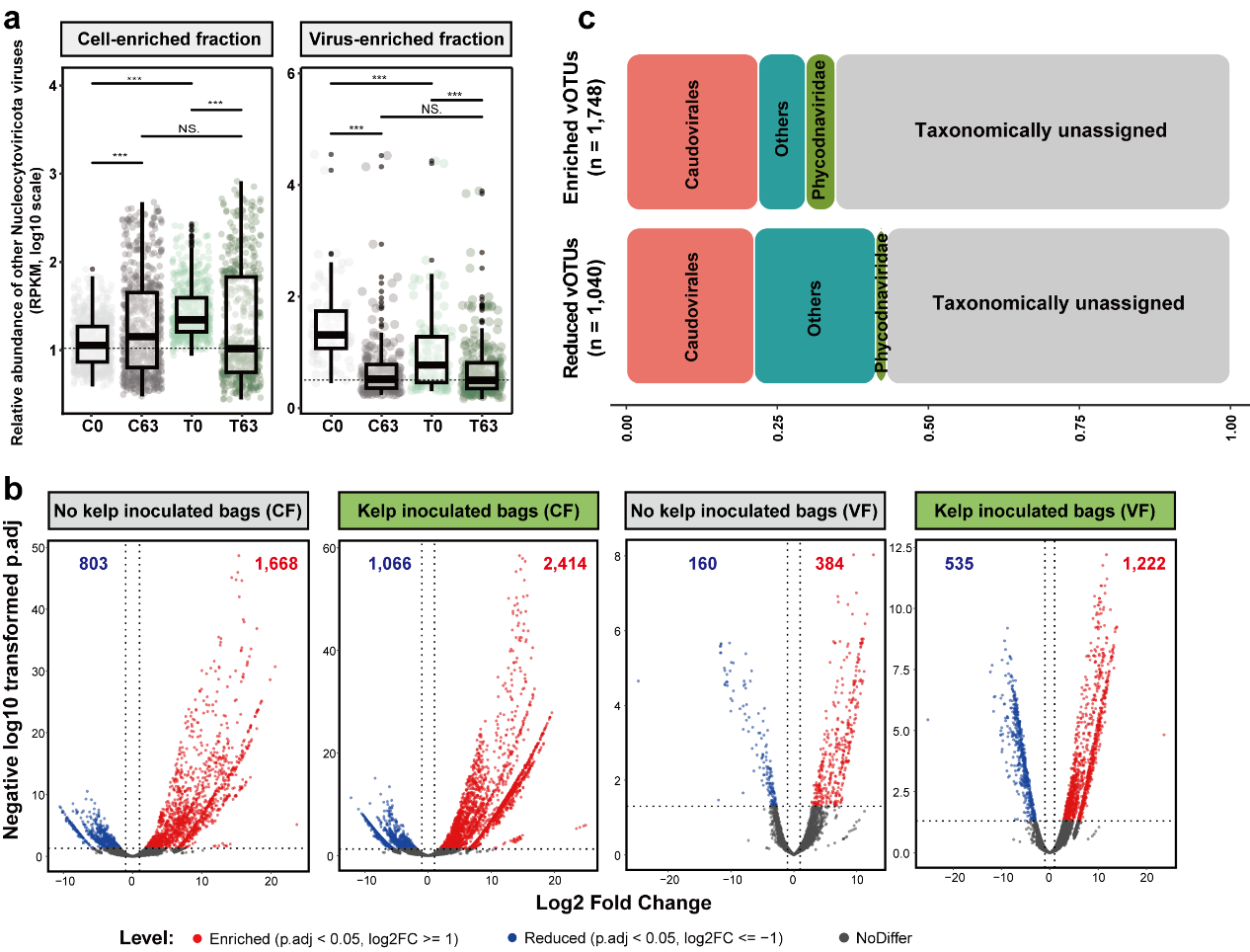


**Fig. S3: Changes in virioplankton communities in the kelp mesocosm experiment. (a)** Changes in the relative abundance of Nucleocytoviricota (not including *Phycodnaviridae*) viruses. **(b)** Volcano plots showing significantly enriched (red dots) and reduced (green dots) viruses in response to kelp cultivation in different fractions of seawater from different mesocosms (with or without kelps). **(c)** Community compositions of significantly enriched and reduced viruses in response to kelp cultivation.


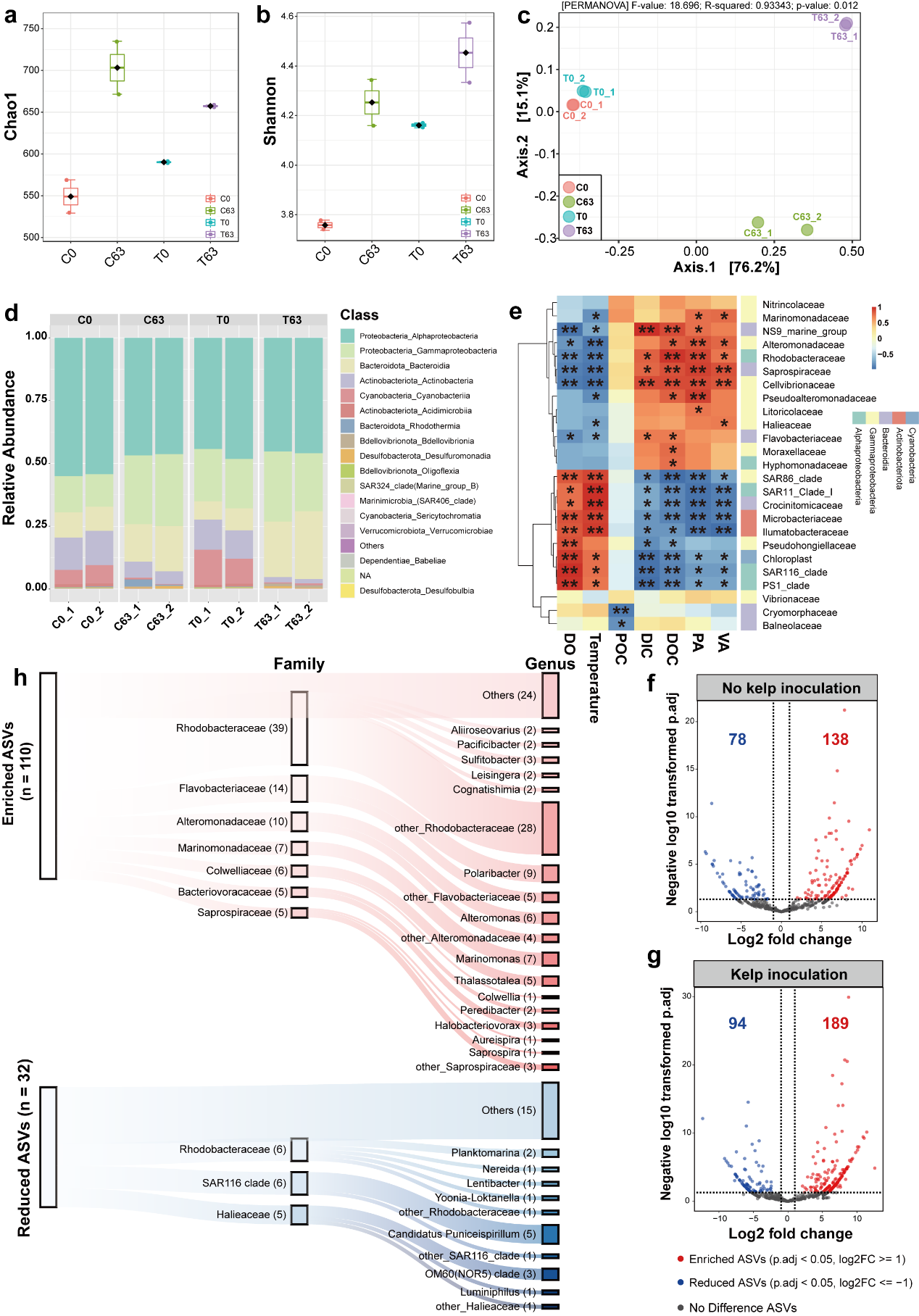


**Fig. S4: Changes in bacterioplankton communities in the kelp mesocosm experiment.** Changes in alpha and beta diversity of bacterioplankton communities, reflected by: **(a)** Chao1 and **(b)** Shannon indexes and **(c)** PCoA. **(d)** Bacterioplankton community compositions at the class level. **(e)** Heatmap showing the correlations between key abiotic and biotic factors (DO, temperature, POC, DIC, DOC, prokaryotic, and viral abundance) and different bacteria groups (at the family level). The volcano plots showing significantly enriched (red dots) and reduced (green dots) bacterial ASVs in response to **(f)** control and **(g)** kelp cultivation. **(h)** Sanky plot showing the taxonomies of the significantly differentially abundant bacteria (ASVs, Left) in response to kelp cultivation. The middle and right bars represent the number of bacterial ASVs in each family and genus, respectively.


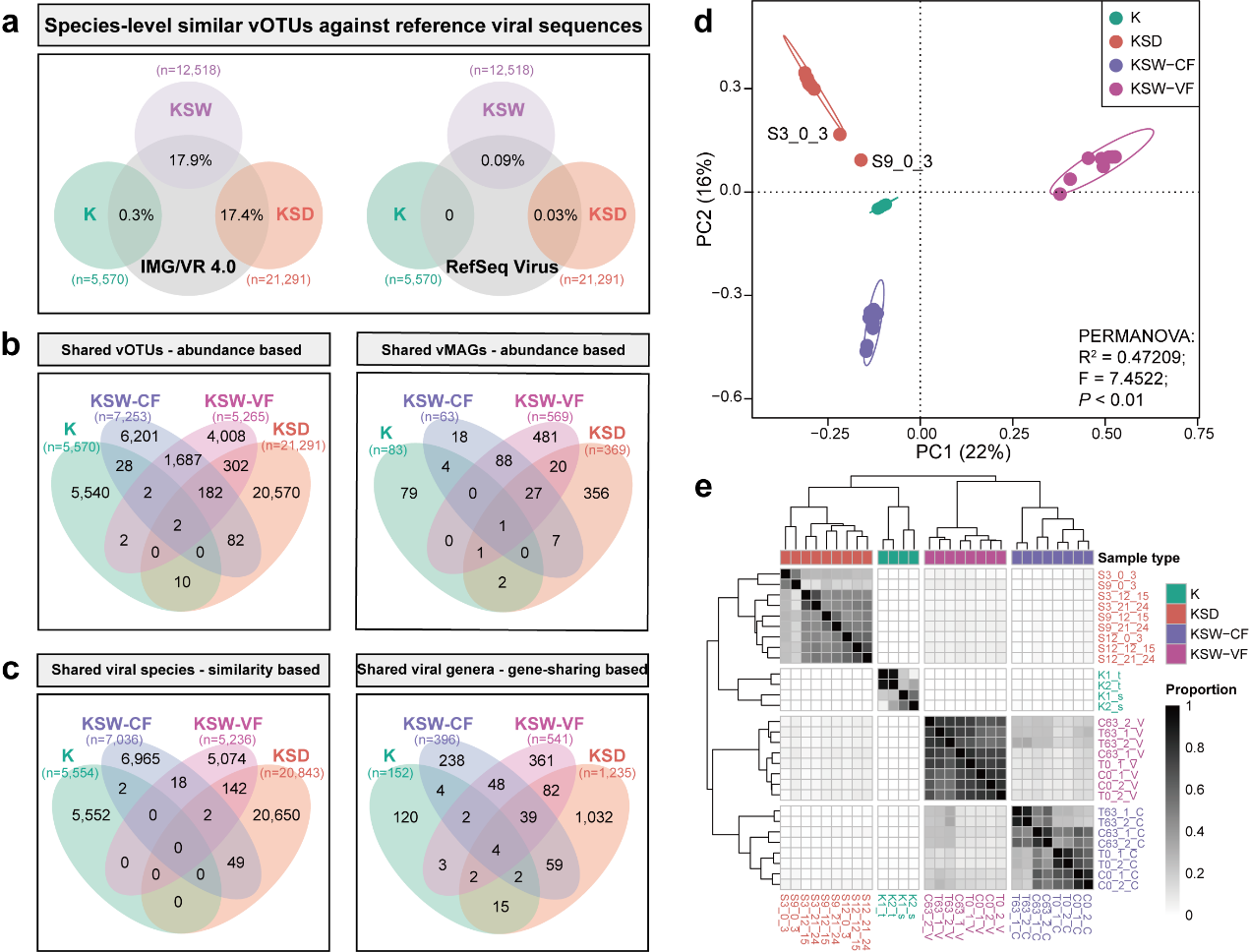


**Fig. S5: Clustering of viruses identified from kelp, seawater, and sediments. (a)** The proportions of viruses similar (at the species level) to reference viral sequences from IMG/VR 4.0 (left) and NCBI RefSeq Virus (right) databases. **(b)** Shared vOTUs (left) and vMAGs (right) across sample types based on the relative abundance (i.e., absence or presence). **(c)** Shared viral species (left) and genera (right) across sample types. **(d)** PCoA analysis of viral communities from different samples. **(e)** Heatmap showing the proportions of shared vOTUs (based on the absence or presence) between different samples.


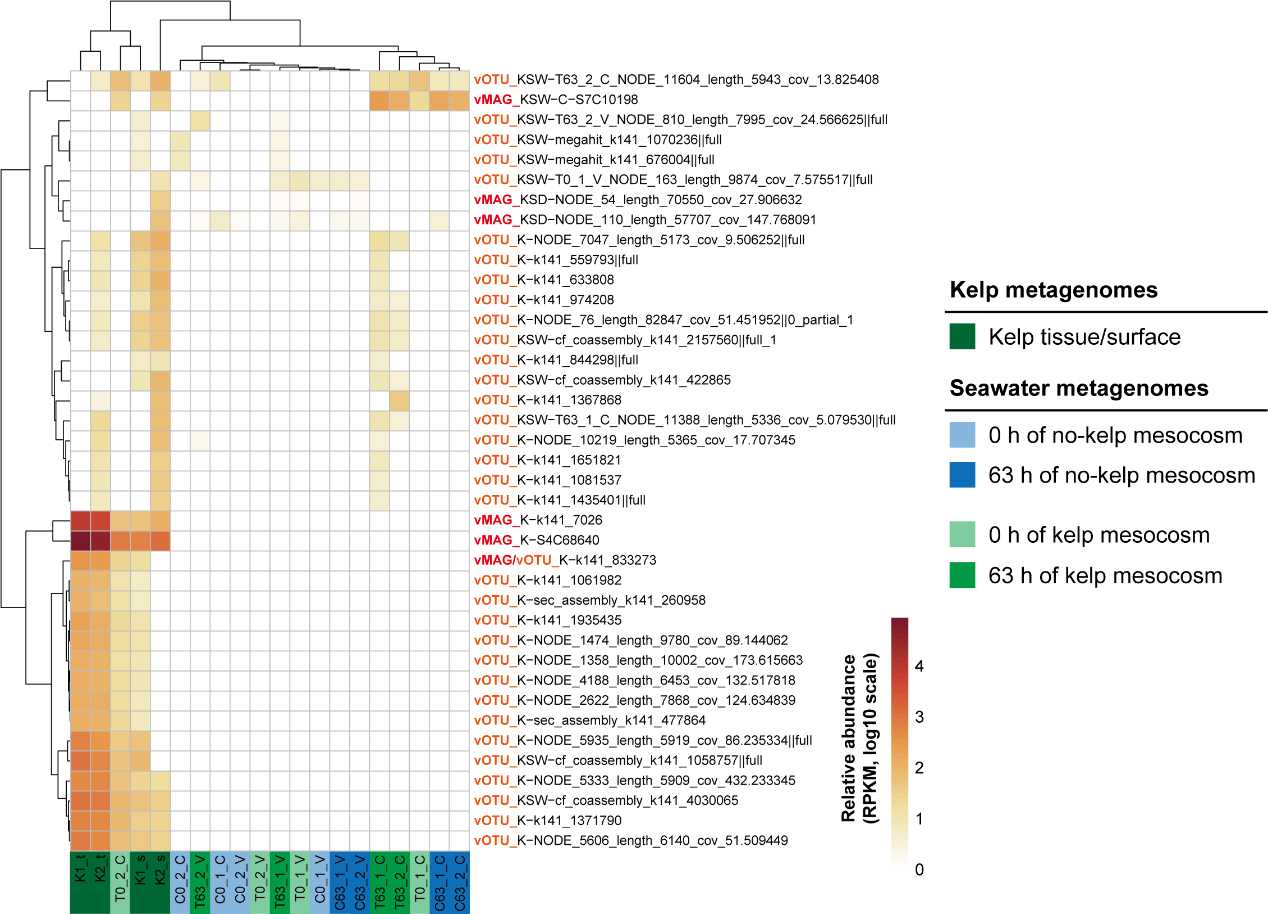


**Fig. S6: Heatmap showing the relative abundance of shared viruses between kelp and seawater samples.**

**
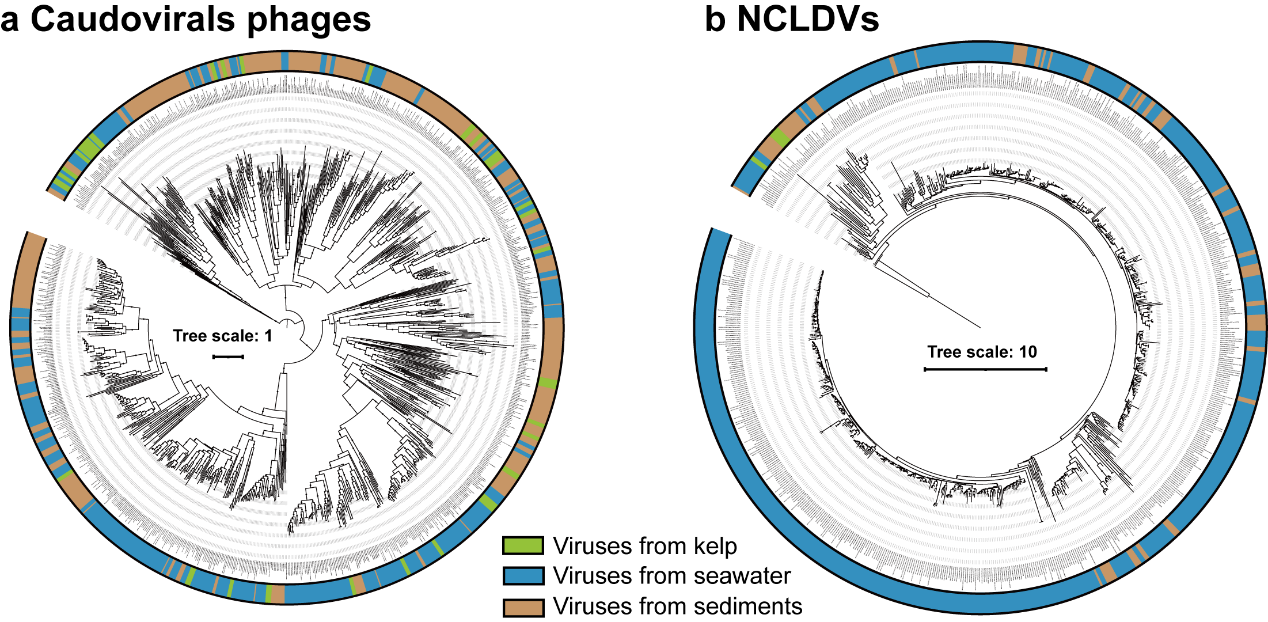
**

**Fig. S7: Maximum-likelihood phylogenetic trees of the (a) Caudovirals phages using terminase large-subunit domains (*terL* genes), and (b) NCLDVs using five highly conserved marker genes of NCLDVs.** Bootstrap values higher than 50% are indicated with a black dot. Viruses originating from kelp, seawater, and sediments are highlighted in green, blue, and brown, respectively.


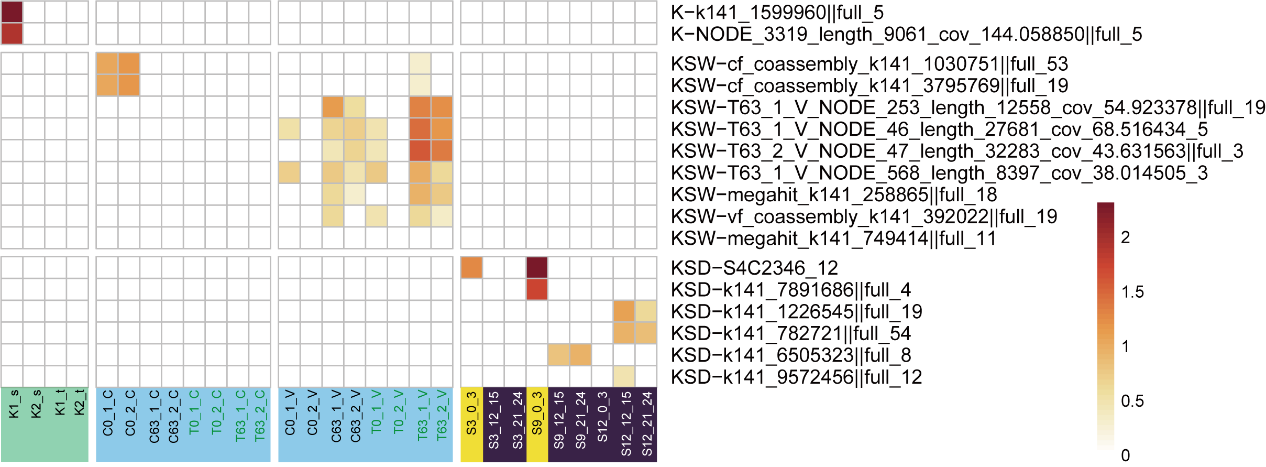


**Fig. S8: Heatmap showing the relative abundance of viruses carrying laminarinase (GH16) genes.**

**
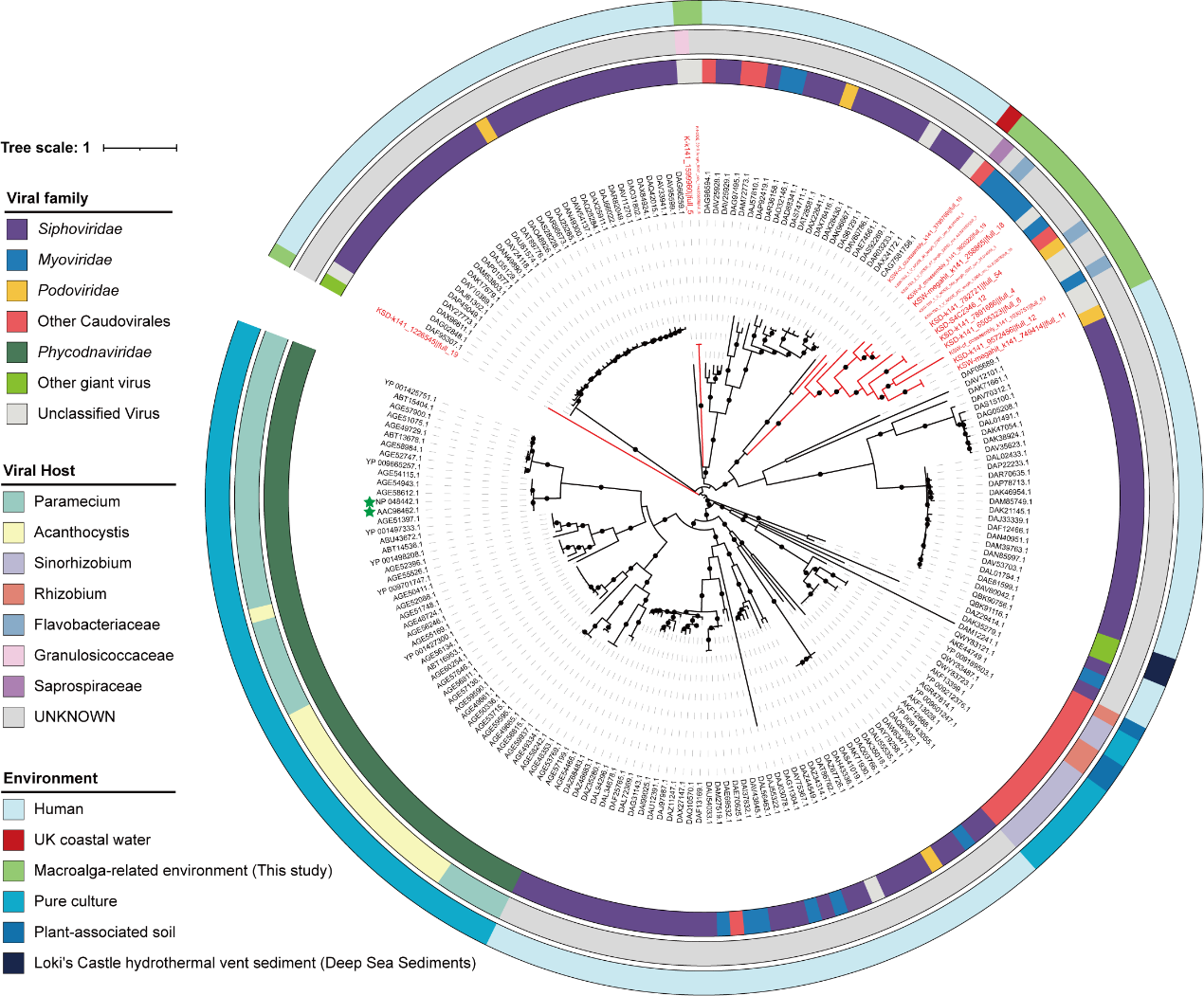
**

**Fig. S9: Maximum-likelihood phylogenetic tree of viral laminarinases.** Seventeen viral laminarinases identified from kelp-associated viruses are colored in red, whereas 186 publicly available viral laminarinases are colored in black. Green asterisks represent the viral laminarinases encoded by a chlorella virus whose ability to hydrolyze the laminarin polymer has been experimentally verified [66]. Bootstrap values higher than 50% are denoted with a black dot., The rings surrounding the tree represent (inside to outside) the viral family, host group, and environment.


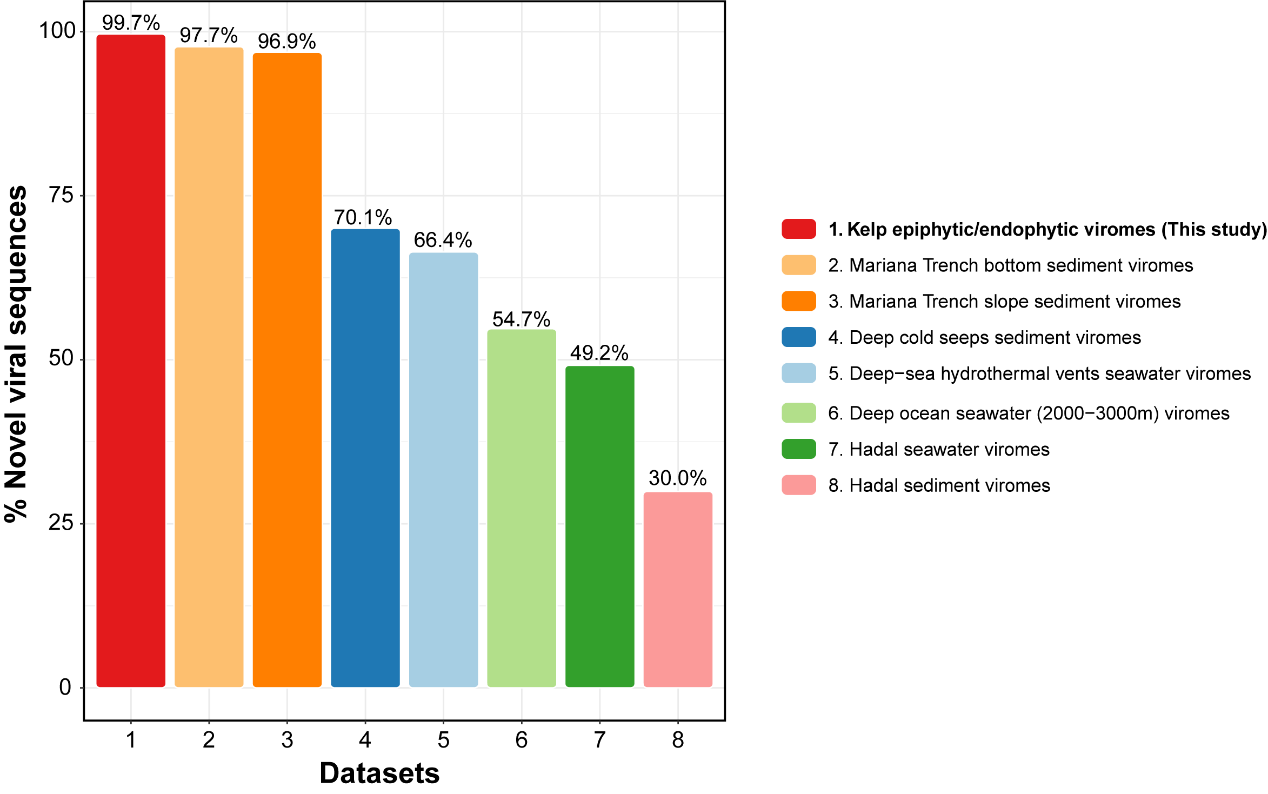


**Fig. S10: Barplot showing the proportions of novel viral species in the virome databases from kelp-associated environments (this study) and other extreme marine environments, including deep ocean[27], hydrothermal vents[67], cold seeps[68], and hadal trenches[69-71].**

**
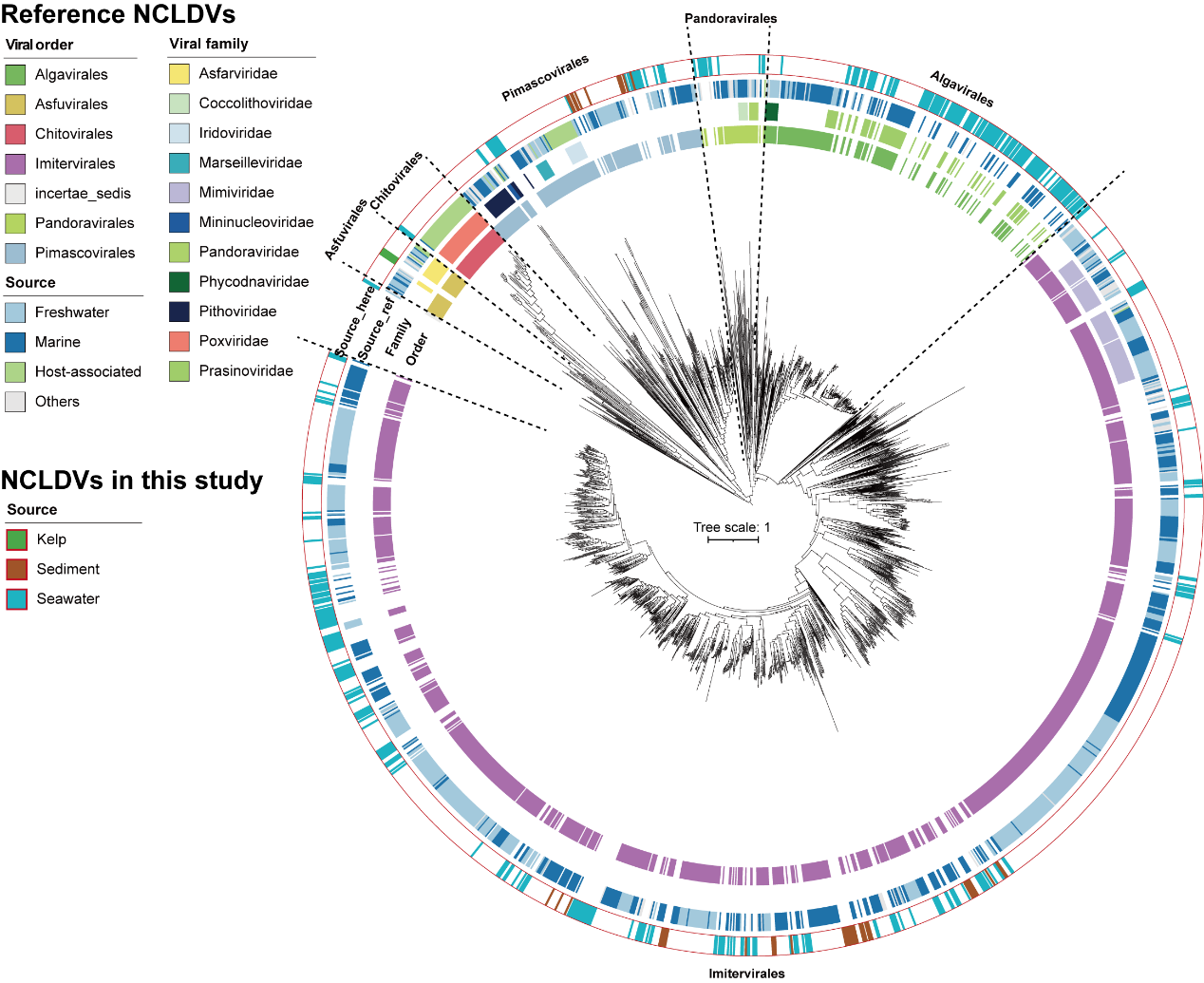
**

**Fig. S11: Phylogenomic tree of Nucleocytoviricota viruses identified from this study (441) and reference viruses retrieved from the GVDB (1,382) based on a concatenated alignment of seven marker genes**. The dendrogram is midpoint rooted. From inside to outside, the concentric rings outside the tree represent the viral order, viral family, source of references, and source of Nucleocytoviricota viruses of this study, respectively. The dashed lines demarcate the different taxonomic groups according to the assigned taxonomies of the reference sequences.

**
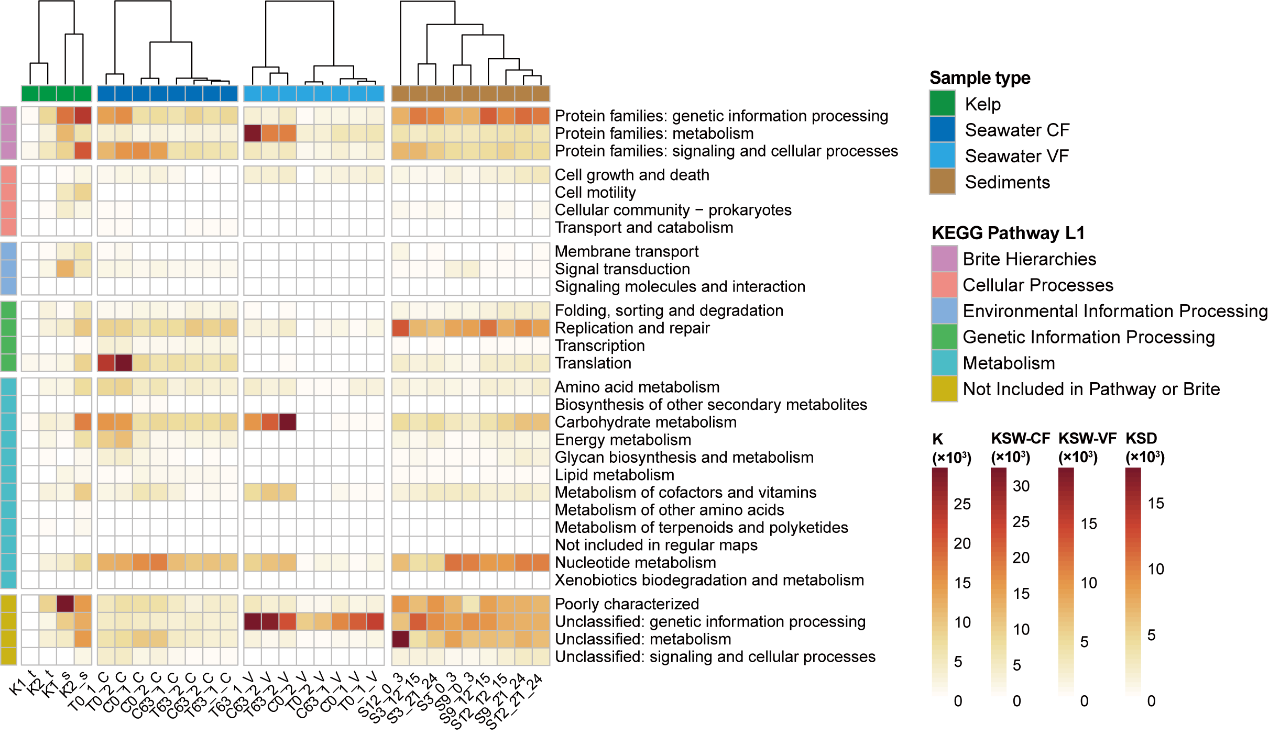
**

**Fig. S12: Heatmap showing the relative abundance of viral functional genes annotated by the KEGG database.**

**
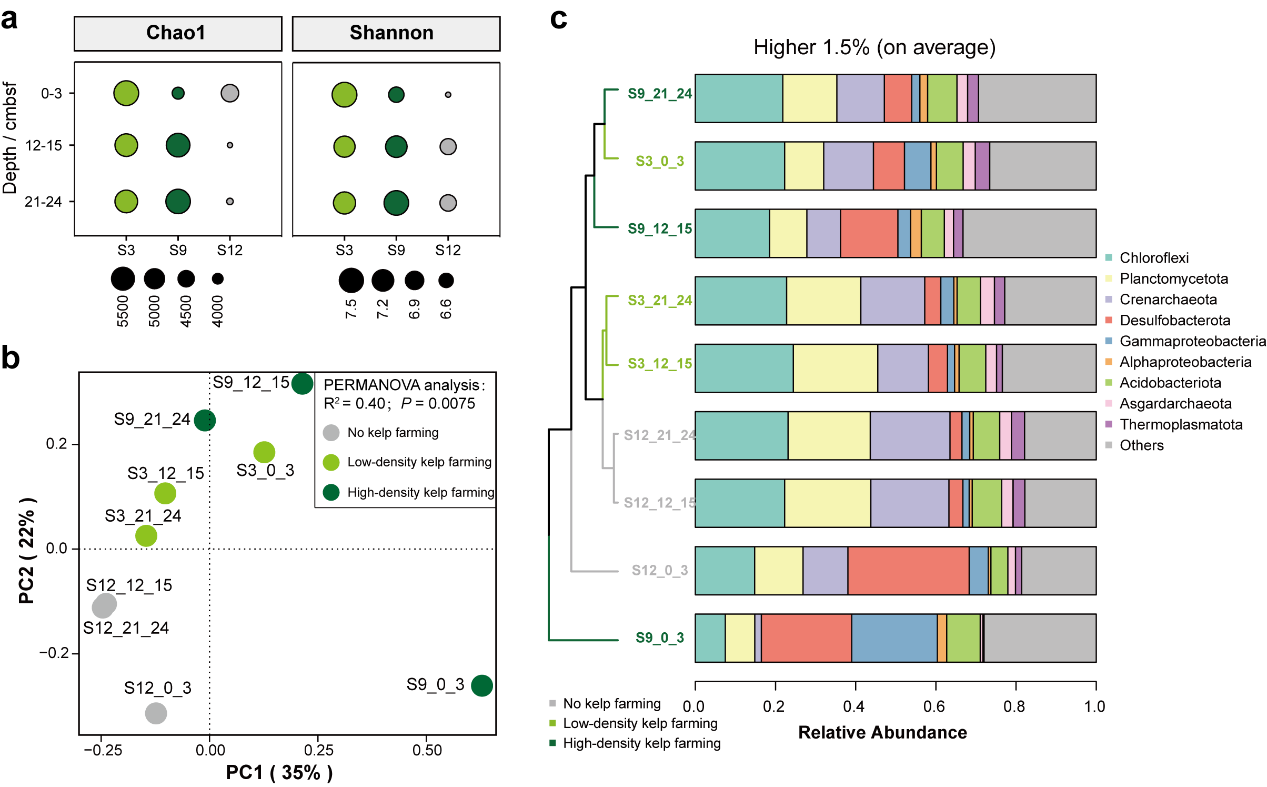
**

**Fig. S13: Prokaryotic community diversity and compositions in kelp-farming sediments. (a)** alpha diversity (Chao1 and Shannon) indexes, **(b)** beta diversity (PCoA and PERMANOVA analyses, and **(c)** community composition at the phylum level (class level for Proteobacteria).


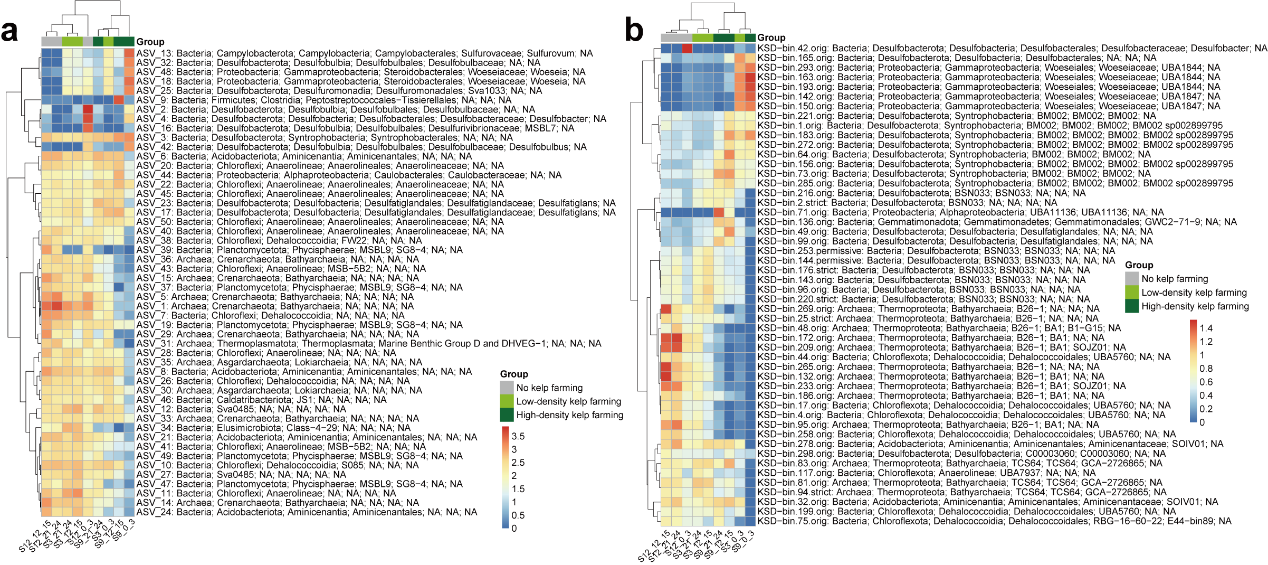


**Fig. S14: Heatmaps showing the relative abundance of the most abundant (top 50) prokaryotic species represented by (a) ASVs and (b) pMAGs.**

**Supplementary Table Legends**

Table S1: Characteristics of all vOTUs identified in this study.

Table S2: Characteristics of all vMAGs identified in this study.

Table S3: Profiles of eukaryotic viruses carrying kelp homologous genes.

Table S4: Summary of homologous viral genes encoded by kelp eukaryotic viruses in the NCBI NR database.

Table S5: Characteristics of all pMAGs identified in this study.

Table S6: Information regarding predicted prokaryotic hosts of viruses (vOTUs and vMAGs).

Table S7: Information regarding prokaryotic metagenome-assembled genomes and draft genomes (retrieved from this study and a previous study) containing prophage regions.

Table S8: Summary of differentially abundant vOTUs in response to kelp cultivation in the mesocosm experiment.

Table S9: Summary of the abiotic and biotic factors of each seawater sample collected from the mesocosm experiment.

Table S10: Results of (partial) mantel tests and the distance-based redundancy analysis (db-RDA) showing the correlations of abiotic factors (DOC, POC, DO) and the viral and prokaryotic communities.

Table S11: Summary of the high-confidence viral AMGs based on DRAM-v annotations.

Table S12: Details about the high-confidence viral AMGs encoding laminarinases.

Table S13: Confidence scores of viral contigs identified by multiple tools and methods.

Table S14: Summary of the prokaryotic ASVs identified from the kelp.

Table S15: Summary of the prokaryotic composition (species level) and their normalized relative abundance calculated from the kelp metagenomes.

Table S16: Summary of the prokaryotic ASVs identified from the mesocosm experiment.

Table S17: Summary of differentially abundant ASVs in response to kelp cultivation in the mesocosm experiment.

Table S18: Summary of the prokaryotic ASVs identified from kelp-farming sediments.

**References**

1 Aramaki T, Blanc-Mathieu R, Endo H, Ohkubo K, Kanehisa M, Goto S et al. KofamKOALA: KEGG Ortholog assignment based on profile HMM and adaptive score threshold. Bioinformatics. 2019;36:2251-2252.

2 King NG, Moore PJ, Thorpe JM, Smale DA. Consistency and variation in the kelp microbiota: Patterns of bacterial community structure across spatial scales. Microb Ecol. 2022;85:1265-1275.

3 Lu D-C, Wang F-Q, Amann RI, Teeling H, Du Z-J. Epiphytic common core bacteria in the microbiomes of co-located green (Ulva), brown (Saccharina) and red (Grateloupia, Gelidium) macroalgae. Microbiome. 2023;11:126.

4 Olga Maria L, Ana Patrícia G. Biofilms: an extra coat on macroalgae. In: Nooruddin T, Dharumadurai D (eds). Algae. IntechOpen: Rijeka (2016) Ch. 8.

5 Cai L, Gao X, Saha M, Han Y, Chang L, Xiao L et al. How do epiphytic and surrounding seawater bacterial communities shift with the development of the Saccharina japonica farmed in the Northern China? Front Mar Sci. 2023;10:1117926.

6 Avcı B, Krüger K, Fuchs BM, Teeling H, Amann RI. Polysaccharide niche partitioning of distinct Polaribacter clades during North Sea spring algal blooms. ISME J. 2020;14:1369-1383.

7 Califano G, Kwantes M, Abreu MH, Costa R, Wichard T. Cultivating the macroalgal holobiont: effects of integrated multi-trophic aquaculture on the microbiome of Ulva rigida (Chlorophyta). Front Mar Sci. 2020;7:52.

8 Dogs M, Wemheuer B, Wolter L, Bergen N, Daniel R, Simon M et al. Rhodobacteraceae on the marine brown alga Fucus spiralis are abundant and show physiological adaptation to an epiphytic lifestyle. Syst Appl Microbiol. 2017;40:370-382.

9 Martin M, Barbeyron T, Martin R, Portetelle D, Michel G, Vandenbol M. The cultivable surface microbiota of the brown alga ascophyllum nodosum is enriched in macroalgal-polysaccharide-degrading bacteria. Front Microbiol. 2015;6:1487.

10 Xie X, He Z, Hu X, Yin H, Liu X, Yang Y. Large-scale seaweed cultivation diverges water and sediment microbial communities in the coast of Nan'ao Island, South China Sea. Sci Total Environ. 2017;598:97-108.

11 Comba González NB, Niño Corredor AN, López Kleine L, Montoya Castaño D. Temporal changes of the epiphytic bacteria community from the marine macroalga Ulva lactuca (Santa Marta, Colombian-Caribbean). Curr Microbiol. 2021;78:534-543.

12 Zhang Q, Fu L, Gui Y, Miao J, Li J. Complete genome sequence of Polaribacter sejongensis NJDZ03 exhibiting diverse macroalgal polysaccharide-degrading activity. Mar Genom. 2022;61:100913.

13 Chafee M, Fernàndez-Guerra A, Buttigieg PL, Gerdts G, Eren AM, Teeling H et al. Recurrent patterns of microdiversity in a temperate coastal marine environment. ISME J. 2018;12:237-252.

14 Rinke C, Chuvochina M, Mussig AJ, Chaumeil P-A, Davín AA, Waite DW et al. A standardized archaeal taxonomy for the Genome Taxonomy Database. Nat Microbiol. 2021;6:946-959.

15 Krumhansl KA, Scheibling RE. Production and fate of kelp detritus. Mar Ecol Prog Ser. 2012;467:281-302.

16 Krause-Jensen D, Duarte CM. Substantial role of macroalgae in marine carbon sequestration. Nat Geosci. 2016;9:737-742.

17 Brunet M, de Bettignies F, Le Duff N, Tanguy G, Davoult D, Leblanc C et al. Accumulation of detached kelp biomass in a subtidal temperate coastal ecosystem induces succession of epiphytic and sediment bacterial communities. Environ Microbiol. 2021;23:1638-1655.

18 Germán AK, Nguyen Dinh L, Le Huu C, Anna F, Andreas K, Le Mai H et al. Bacterial community composition of the sea grape Caulerpa lentillifera: a comparison between healthy and diseased states. bioRxiv. 2021:2021.2006.2030.450479.

19 Sun Y, Song Z, Zhang H, Liu P, Hu X. Seagrass vegetation affect the vertical organization of microbial communities in sediment. Mar Environ Res. 2020;162:105174.

20 Emil Ruff S, Probandt D, Zinkann A-C, Iversen MH, Klaas C, Würzberg L et al. Indications for algae-degrading benthic microbial communities in deep-sea sediments along the Antarctic Polar Front. Deep-Sea Res Pt II. 2014;108:6-16.

21 Mussmann M, Pjevac P, Kruger K, Dyksma S. Genomic repertoire of the Woeseiaceae/JTB255, cosmopolitan and abundant core members of microbial communities in marine sediments. ISME J. 2017;11:1276-1281.

22 Corrigan S, Brown AR, Ashton IGC, Smale DA, Tyler CR. Quantifying habitat provisioning at macroalgal cultivation sites. Rev Aquac. 2022;14:1671-1694.

23 Xiong T, Li H, Hu Y, Zhai W-d, Zhang Z, Liu Y et al. Seaweed farming environments do not always function as CO_2_ sink under synergistic influence of macroalgae and microorganisms. Agric, Ecosyst Environ. 2024;361:108824.

24 Li H, Zhang Z, Xiong T, Tang K, He C, Shi Q et al. Carbon sequestration in the form of recalcitrant dissolved organic carbon in a seaweed (kelp) farming environment. Environ Sci Technol. 2022;56:9112-9122.

25 Zhang Z, Hu Y-B. Assessment on seasonal acidification and its controls in the Muping Marine Ranch, Yantai, China. Mar Pollut Bull. 2023;189:114826.

26 Feng X, Li H, Zhang Z, Xiong T, Shi X, He C et al. Microbial-mediated contribution of kelp detritus to different forms of oceanic carbon sequestration. Ecol Indicators. 2022;142:109186.

27 Zhao J, Wang Z, Li C, Shi T, Liang Y, Jiao N et al. Significant differences in planktonic virus communities between “cellular fraction” (0.22 ~ 3.0 µm) and “viral fraction” (< 0.22 μm) in the ocean. Microb Ecol. 2022;86:825-842.

28 Chen J, Li H, Zhang Z, He C, Shi Q, Jiao N et al. DOC dynamics and bacterial community succession during long-term degradation of Ulva prolifera and their implications for the legacy effect of green tides on refractory DOC pool in seawater. Water Res. 2020;185:116268.

29 Callahan BJ, McMurdie PJ, Rosen MJ, Han AW, Johnson AJA, Holmes SP. DADA2: High-resolution sample inference from Illumina amplicon data. Nat Methods. 2016;13:581-583.

30 Chong J, Liu P, Zhou G, Xia J. Using MicrobiomeAnalyst for comprehensive statistical, functional, and meta-analysis of microbiome data. Nat Protoc. 2020;15:799-821.

31 Nair S, Zhang Z, Li H, Zhao H, Shen H, Kao S-J et al. Inherent tendency of Synechococcus and heterotrophic bacteria for mutualism on long-term coexistence despite environmental interference. Sci Adv. 2022;8:eabf4792.

32 Wood DE, Lu J, Langmead B. Improved metagenomic analysis with Kraken 2. Genome Biol. 2019;20:257.

33 Lu J, Breitwieser FP, Thielen P, Salzberg SL. Bracken: estimating species abundance in metagenomics data. PeerJ Comput Sci. 2017;3:e104.

34 Lu J, Rincon N, Wood DE, Breitwieser FP, Pockrandt C, Langmead B et al. Metagenome analysis using the Kraken software suite. Nat Protoc. 2022;17:2815-2839.

35 Kang DD, Li F, Kirton E, Thomas A, Egan R, An H et al. MetaBAT 2: an adaptive binning algorithm for robust and efficient genome reconstruction from metagenome assemblies. PeerJ. 2019;7:e7359.

36 Wu YW, Simmons BA, Singer SW. MaxBin 2.0: an automated binning algorithm to recover genomes from multiple metagenomic datasets. Bioinformatics. 2016;32:605-607.

37 Alneberg J, Bjarnason BS, de Bruijn I, Schirmer M, Quick J, Ijaz UZ et al. Binning metagenomic contigs by coverage and composition. Nat Methods. 2014;11:1144-1146.

38 Uritskiy GV, DiRuggiero J, Taylor J. MetaWRAP-a flexible pipeline for genome-resolved metagenomic data analysis. Microbiome. 2018;6:158.

39 Olm MR, Brown CT, Brooks B, Banfield JF. dRep: a tool for fast and accurate genomic comparisons that enables improved genome recovery from metagenomes through de-replication. ISME J. 2017;11:2864-2868.

40 Nayfach S, Roux S, Seshadri R, Udwary D, Varghese N, Schulz F et al. A genomic catalog of Earth's microbiomes. Nat Biotechnol. 2021;39:499-509.

41 Coclet C, Roux S. Global overview and major challenges of host prediction methods for uncultivated phages. Curr Opin Virol. 2021;49:117-126.

42 von Meijenfeldt FAB, Arkhipova K, Cambuy DD, Coutinho FH, Dutilh BE. Robust taxonomic classification of uncharted microbial sequences and bins with CAT and BAT. Genome Biol. 2019;20:217.

43 Chaumeil PA, Mussig AJ, Hugenholtz P, Parks DH. GTDB-Tk: a toolkit to classify genomes with the Genome Taxonomy Database. Bioinformatics. 2019;36:1925-1927.

44 Johansen J, Plichta DR, Nissen JN, Jespersen ML, Shah SA, Deng L et al. Genome binning of viral entities from bulk metagenomics data. Nat Commun. 2022;13:965.

45 Nayfach S, Camargo AP, Schulz F, Eloe-Fadrosh E, Roux S, Kyrpides NC. CheckV assesses the quality and completeness of metagenome-assembled viral genomes. Nat Biotechnol. 2021;39:578-585.

46 Tettelin H, Masignani V, Cieslewicz MJ, Donati C, Medini D, Ward NL et al. Genome analysis of multiple pathogenic isolates of Streptococcus agalactiae: Implications for the microbial “pan-genome”. Proc Natl Acad Sci USA. 2005;102:13950-13955.

47 Pratama AA, Bolduc B, Zayed AA, Zhong Z-P, Guo J, Vik DR et al. Expanding standards in viromics: in silico evaluation of dsDNA viral genome identification, classification, and auxiliary metabolic gene curation. PeerJ. 2021;9:e11447.

48 Bin Jang H, Bolduc B, Zablocki O, Kuhn JH, Roux S, Adriaenssens EM et al. Taxonomic assignment of uncultivated prokaryotic virus genomes is enabled by gene-sharing networks. Nat Biotechnol. 2019;37:632-639.

49 Pons JC, Paez-Espino D, Riera G, Ivanova N, Kyrpides NC, Llabrés M. VPF-Class: taxonomic assignment and host prediction of uncultivated viruses based on viral protein families. Bioinformatics. 2021;37:1805-1813.

50 Aylward FO, Moniruzzaman M. ViralRecall-a flexible command-line tool for the detection of giant virus signatures in 'omic data. Viruses. 2021;13:150.

51 Schulz F, Roux S, Paez-Espino D, Jungbluth S, Walsh DA, Denef VJ et al. Giant virus diversity and host interactions through global metagenomics. Nature. 2020;578:432-436.

52 Xia R, Sun M, Balcázar JL, Yu P, Hu F, Alvarez PJJ. Benzo[a]pyrene stress impacts adaptive strategies and ecological functions of earthworm intestinal viromes. ISME J. 2023;17:1004-1014.

53 Shang J, Tang X, Sun Y. PhaTYP: predicting the lifestyle for bacteriophages using BERT. Brief Bioinform. 2022;24:bbac487.

54 Moniruzzaman M, Martinez-Gutierrez CA, Weinheimer AR, Aylward FO. Dynamic genome evolution and complex virocell metabolism of globally-distributed giant viruses. Nat Commun. 2020;11:1710.

55 Aylward FO, Moniruzzaman M, Ha AD, Koonin EV. A phylogenomic framework for charting the diversity and evolution of giant viruses. PLoS Biol. 2021;19:e3001430.

56 Price MN, Dehal PS, Arkin AP. FastTree 2--approximately maximum-likelihood trees for large alignments. PLoS One. 2010;5:e9490.

57 Letunic I, Bork P. Interactive Tree Of Life (iTOL) v5: an online tool for phylogenetic tree display and annotation. Nucleic Acids Res. 2021;49:W293-W296.

58 Adriaenssens EM, Cowan DA. Using signature genes as tools to assess environmental viral ecology and diversity. Appl Environ Microbiol. 2014;80:4470-4480.

59 Weinheimer AR, Aylward FO. Infection strategy and biogeography distinguish cosmopolitan groups of marine jumbo bacteriophages. ISME J. 2022;16:1657-1667.

60 Katoh K, Standley DM. MAFFT multiple sequence alignment software version 7: improvements in performance and usability. Mol Biol Evol. 2013;30:772-780.

61 Capella-Gutiérrez S, Silla-Martínez JM, Gabaldón T. trimAl: a tool for automated alignment trimming in large-scale phylogenetic analyses. Bioinformatics. 2009;25:1972-1973.

62 Nguyen L-T, Schmidt HA, von Haeseler A, Minh BQ. IQ-TREE: A fast and rffective stochastic algorithm for estimating maximum-likelihood phylogenies. Mol Biol Evol. 2014;32:268-274.

63 Kalyaanamoorthy S, Minh BQ, Wong TKF, von Haeseler A, Jermiin LS. ModelFinder: fast model selection for accurate phylogenetic estimates. Nat Methods. 2017;14:587-589.

64 Di Salvo M, Puccio S, Peano C, Lacour S, Alifano P. RhoTermPredict: an algorithm for predicting Rho-dependent transcription terminators based on Escherichia coli, Bacillus subtilis and Salmonella enterica databases. BMC Bioinformatics. 2019;20:117.

65 Lu S, Wang J, Chitsaz F, Derbyshire MK, Geer RC, Gonzales NR et al. CDD/SPARCLE: the conserved domain database in 2020. Nucleic Acids Res. 2020;48:D265-D268.

66 Sun L, Gurnon JR, Adams BJ, Graves MV, Van Etten JL. Characterization of a β-1,3-glucanase encoded by Chlorella virus PBCV-1. Virology. 2000;276:27-36.

67 Cheng R, Li X, Jiang L, Gong L, Geslin C, Shao Z. Virus diversity and interactions with hosts in deep-sea hydrothermal vents. Microbiome. 2022;10:235.

68 Li Z, Pan D, Wei G, Pi W, Zhang C, Wang JH et al. Deep sea sediments associated with cold seeps are a subsurface reservoir of viral diversity. ISME J. 2021;15:2366-2378.

69 Jian H, Yi Y, Wang J, Hao Y, Zhang M, Wang S et al. Diversity and distribution of viruses inhabiting the deepest ocean on Earth. ISME J. 2021;15:3094-3110.

70 Zhao J, Jing H, Wang Z, Wang L, Jian H, Zhang R et al. Novel viral communities potentially assisting in carbon, nitrogen, and sulfur metabolism in the upper slope sediments of Mariana Trench. mSystems. 2022;7:e01358-01321.

71 Zhou Y-L, Mara P, Vik D, Edgcomb VP, Sullivan MB, Wang Y. Ecogenomics reveals viral communities across the Challenger Deep oceanic trench. Commun Biol. 2022;5:1055.
